# Thermophysiological and perceptual responses to exercise heat stress in people with cystic fibrosis on Elexacaftor/Tezacaftor/Ivacaftor

**DOI:** 10.1113/EP093605

**Published:** 2026-09-12

**Authors:** Lauren J. Clayton, Jo Corbett, Anthony I. Shepherd, Heather Massey, Don S. Urquhart, Neil Maxwell, Gary Connett, Mark Allenby, Julian Legg, Thomas Daniels, Zoe L. Saynor

**Affiliations:** ^1^ Clinical, Health and Rehabilitation Research Team, School of Psychology, Sport and Health Science, Faculty of Science and Health University of Portsmouth Portsmouth UK; ^2^ Extreme Environments and Occupational Performance Research Group, School of Psychology, Sport and Health Science, Faculty of Science and Health University of Portsmouth Portsmouth UK; ^3^ University Hospital Southampton NHS Foundation Trust Southampton UK; ^4^ Department of Paediatric Respiratory and Sleep Medicine Royal Hospital for Children and Young People Edinburgh Scotland UK; ^5^ Department of Child Life and Health University of Edinburgh Edinburgh Scotland UK; ^6^ Environmental Extremes Laboratory University of Brighton Eastbourne UK; ^7^ Southampton Biomedical Research Centre National Institute for Health Research Southampton UK; ^8^ School of Health Sciences Faculty of Environmental and Life Sciences University of Southampton Southampton UK

**Keywords:** exertional heat illness, heat stress, respiratory disease, thermal physiology

## Abstract

The objective of this study was to investigate thermophysiological and perceptual responses to exercise heat stress in people with cystic fibrosis (pwCF) on Elexacaftor/Tezacaftor/Ivacaftor (ETI) versus healthy controls, and to explore their lived experiences of heat stress. This mixed‐methods, multi‐study project comprised: (i) a laboratory study where pwCF (*n *= 6) and matched controls (*n *= 6) completed two 30 minutes cycling bouts in the heat (35°C, 30% relative humidity), at 150 and 250 W·m^−2^ metabolic heat production. Measures included sweat sodium chloride ([Na^+^Cl^−^]), local and whole body sweat rate, deep body and skin temperatures, heart rate (HR), forearm and finger skin blood flow (SkBF), and perceptual responses (exertion, thermal sensation and comfort, thirst, heat‐illness symptom index (HISI)); and (ii) a pilot e‐survey (*n *= 30) exploring free‐living sweating, heat experiences, hydration and salt‐supplementation practices. Laboratory data showed no significant differences in sweat [Na^+^Cl^−^] (CON: 45 (36–106) mmol·L^−1^; pwCF: 75 (59–92) mmol·L^−1^, *P *= 0.45), sweating rate, deep body and skin temperature, HR, SkBF, perceived exertion, thermal comfort, thermal sensation, or HISI (*P *> 0.05). However, pwCF reported greater thirst at baseline (*P *= 0.045) and after 30 minutes cycling (*P *= 0.01), despite comparable hydration status. Survey responses indicated some pwCF perceive reduced sweating since starting ETI, yet their salt supplementation is unchanged. PwCF treated with ETI show thermophysiological/perceptual responses equivalent to healthy individuals during exercise heat stress. Together with survey insights, this suggests ETI may reduce previous heat‐related vulnerabilities and better prepare pwCF for exercise in hot conditions. Updated evidence is needed to refine salt‐replacement and hydration strategies, modernise clinical guidance, and support safe physical activity in a warming climate.

## Abstract (highlights)

- **What is the central question of this study?**
  What are the thermophysiological and perceptual responses to controlled exercise heat stress in people with cystic fibrosis on cystic fibrosis transmembrane conductance regulator modulator therapy versus healthy individuals, and what are their lived experiences of heat, hydration and salt intake practices?
- **What is the main finding and its importance?**
  People with cystic fibrosis showed thermophysiological and perceptual responses similar to healthy controls but reported greater thirst and dryness, though most have not altered salt supplementation/hydration practices. This highlights the need for evolving, physiologically informed hydration and electrolyte guidance, particularly for exercise in the heat.

## 1 INTRODUCTION

Global temperatures are rising at an unprecedented rate. The year 2024 was the warmest on record, continuing a pattern in which the 10 warmest years have all occurred since 2014 (NOAA National Centers for Environmental Information, 2024). Anthropogenic climate change is also driving more frequent and extreme heatwaves (AghaKouchak et al., 2020), posing significant health risks for populations who may be particularly vulnerable to heat stress (Tipton et al., 2023). Understanding how specific clinical groups respond to heat is therefore a growing public health priority. People with cystic fibrosis (pwCF) may be uniquely vulnerable to heat, owing to well‐established disturbances in sweat gland physiology (Quinton, 1983) and, potentially, thermoregulation (Bar‐Or et al., 1992; Kriemler et al., 1999; Orenstein et al., 1983).

In healthy individuals, the CF transmembrane conductance regulator (CFTR) protein functions as a chloride (Cl^−^) ion channel in eccrine sweat glands and regulates the epithelial sodium (Na^+^) channel, allowing effective reabsorption of sodium and chloride during sweating (Saint‐Criq & Gray, 2017). Prior to the advent of CFTR modulator therapy, dysfunction of this process in pwCF resulted in markedly elevated sweat Na^+^Cl^−^ concentrations; up to 3–5 times higher than in healthy individuals (Emrich et al., 1968). Traditionally, excessive salt loss has predisposed pwCF to hyponatraemia (Ballestero et al., 2006; Sawka et al., 2009), dehydration (Bar‐Or et al., 1992) which can reduce sweating (Périard et al., 2021), and reduced evaporative heat loss due to salt accumulation on the skin (Taylor & Machado‐Moreira, 2013), all of which can compromise thermoregulation and increase susceptibility to heat‐related illness.

Evaporative heat loss is supported by cutaneous vasodilation, which increases skin blood flow and facilitates heat transfer from the body core to the skin, enabling heat dissipation to the environment (Saint‐Criq & Gray, 2017). However, vascular endothelial dysfunction has been reported in pwCF before (Rodriguez‐Miguelez et al., 2016) and persisting after initiation of CFTR modulators (Clayton et al., 2025), which may impair skin blood flow and reduce heat transfer to the skin. When combined with altered sweating responses, these vascular abnormalities may increase vulnerability to thermal strain (Kessler & Andersen, 1951; Williams et al., 1976) and heat illness risk in pwCF (Sawka et al., 1992), particularly during prolonged exercise or environmental heat stress (Orenstein et al., 1983, 1984).

Despite this theoretical vulnerability and the growing urgency posed by climate change and importance of regular physical activity and exercise for pwCF (Gruet et al., 2022; Radtke et al., 2022; Southern et al., 2024), only early seminal investigations conducted over 40 years ago have comprehensively examined thermoregulatory responses during prolonged exercise in the heat in this population (Orenstein et al., 1983, 1984). Orenstein et al. reported elevated sweat [Na^+^Cl^−^] in pwCF compared to healthy controls, but no wider thermoregulatory differences in the heat (Orenstein et al., 1983, 1984). However, their interpretation is constrained by methodological limitations. The techniques available at the time did not permit a comprehensive evaluation of thermoregulatory responses to heat stress, and they did not control for factors now known to strongly influence heat responses, such as age (Blatteis, 2012), sex (Gagnon et al., 2008), height, weight, body surface area (BSA) (Cramer & Jay, 2015) and metabolic heat production (MHP) (Gagnon et al., 2008). Moreover, these findings predate the transformative era of CFTR modulator therapies.

CFTR modulators, particularly Elexacaftor/Tezacaftor/Ivacaftor (ETI), have fundamentally reshaped the physiological and behavioural landscape of CF. These therapies improve the production and function of CFTR protein (Lopes‐Pacheco, 2020), significantly reducing sweat [Cl^−^] at rest (McKone et al., 2014; Ramsey et al., 2011; Taylor‐Cousar et al., 2017), and potentially altering electrolyte loss during exercise and heat exposure, although this has not been investigated. For the first time, pwCF may be losing less salt in their sweat, raising critical questions about whether salt‐supplementation recommendations (Wilschanski et al., 2024) remain appropriate. At the same time, improvements in respiratory health, wellbeing and exercise capacity have enabled many pwCF to engage in more vigorous physical activity and spend more time outdoors, contexts associated with greater heat exposure. Yet emerging evidence suggests that not all physiological systems normalise with ETI; for example, persisting vascular endothelial abnormalities (Clayton et al., 2025). Together, these changes create a fundamentally new thermophysiological landscape for pwCF. Given rising global temperatures, increased exercise participation, and the absence of contemporary data describing thermoregulation or hydration needs in pwCF in the modulator era, there is an urgent need for modern, mechanistic and contextually relevant research.

Therefore, this study aimed to: (1) compare thermophysiological and perceptual responses during prolonged cycling at a fixed metabolic heat production in the heat between pwCF on ETI and matched healthy controls; (2) explore lived experiences of heat illness, dehydration symptoms and sweating, particularly since initiating CFTR modulator therapy; and (3) assess the use of salt supplements and fluid requirements at rest, during exercise and in heat in pwCF.

## 2 METHODS

### 2.1 Ethical approval

This multi‐experiment paper comprised (1) a mechanistic laboratory‐based study, and (2) an exploratory, pilot e‐survey study. The laboratory study was pre‐registered on ClincalTrials.gov (NCT05896488), received a favourable ethical opinion from the South East Scotland NHS Research Ethics Committee 01 (22/SS/0102) and UK Health Research Authority, and adhered to the *Declaration of Helsinki*. Written informed consent was obtained from all adult participants, while assent was provided by paediatric participants, alongside caregiver consent. Data collection took place in the Extreme Environments Laboratories at the University of Portsmouth, UK.

Favourable ethics opinion for the exploratory, pilot e‐survey study was obtained from the Faculty of Science and Health Ethics Committee, University of Portsmouth (SFHEC 2023–060). Participants were provided with an information sheet and electronic consent form, with consent obtained via a tick box preceding the survey content. Withdrawal was possible at any time, by exiting the survey, with incomplete responses excluded. Voluntary, anonymous responses were collected using an online survey platform (JISC, Bristol, UK) between July 2023 and July 2024.

### 2.2 Participants

For the laboratory study, an a priori power calculation, based on previously reported differences in sweat [Na^+^] during cycling exercise in the heat between pwCF and healthy controls (Orenstein et al., 1983), determined that *n *= 12 (6/group) was required to achieve 80% power at α = 0.05, to detect a significant post‐exercise difference in the primary outcome measure (sweat [Na^+^]).

A total of six pwCF (4 adults, 2 children/adolescents; Table 1) and six healthy age‐, sex‐, height‐ and weight‐matched controls participated in the laboratory study (Table 1). Inclusion criteria for pwCF included a confirmed CF diagnosis (clinical features and abnormal sweat Cl^−^ > 60 mmol·L^−1^ prior to initiating treatment with CFTR modulators), being stable on ETI, age ≥10 years, body mass ≥ 40 kg, and no symptom exacerbation or weight loss in the preceding two weeks. Exclusion criteria included unstable co‐morbid asthma (daily pulmonary function variability >20%), any non‐pulmonary conditions that may impair exercise ability, a smoker, pregnant or using vasoactive medications. Healthy controls were recruited individually, after each participant with CF, to ensure they were matched for age, sex, height and weight. Controls were required to be free from chronic disease, non‐smokers, not pregnant and not contraindicated to exhaustive exercise.

**TABLE 1:** Baseline clinical characteristic for the cystic fibrosis group and anthropometric, pulmonary function and aerobic fitness for participants with cystic fibrosis and healthy age‐, sex‐, height‐ and weight‐matched control participants upon initiation into the laboratory‐based study.

Variable	CON (*n *= 6)	pwCF (*n *= 6)	*P*	*ES*
Months on ETI	—	32.7 ± 10.4	—	—
Genotype (*n*)				
∆F508 Homozygote	—	3	—	—
∆F508 Heterozygote	—	3	—	—
Pancreatic insufficient (*n*)	—	6	—	—
CF‐related diabetes (*n*)	—	0	—	—
CF‐related liver disease (*n*)	—	1	—	—
Sex (F/M)	1/5	1/5	—	—
Age (years)	31.6 ± 17.8	34.0 ± 17.1	0.82	0.14
Stature (m)	1.73 ± 1.42	1.73 ± 1.11	0.97	0.03
Weight (kg)	71.1 ± 23.4	68.8 ± 18.7	0.85	0.11
BSA (m^2^)	1.84 ± 0.36	1.81 ± 0.28	0.91	0.07
SSA (cm^2^·kg^−1^)	267.9 ± 35.6	271.7 ± 36.9	0.86	0.11
BMI (kg·m^−2^)	23.2 ± 4.6	22.6 ± 4.2	0.81	0.14
Lung function				
FVC (L)	4.49 ± 0.86	4.53 ± 1.32	0.95	0.04
FVC (% predicted)^a^	94.2 ± 7.5	94.0 ± 15.5	0.87	0.10
FVC (*z*‐score)	−0.38 ± 0.60	−0.56 ± 1.23	0.76	0.18
FEV_1_ (L)	3.44 ± 0.62	3.44 ± 0.84	0.99	0.01
FEV_1_ (% predicted)^a^	88.2 ± 5.8	87.5 ± 15.1	0.92	0.06
FEV_1_ (*z*‐score)	−0.93 ± 0.40	−1.06 ± 1.17	0.81	0.14
FEF_25–75%_ (%)	2.85 ± 0.79	2.82 ± 0.68	0.95	0.04
FEF_25–75%_ (% predicted)^a^	70.3 ± 10.6	72.5 ± 22.0	0.83	0.13
FEF_25–75%_ (*z*‐score)	−1.19 ± 0.37	−1.22 ± 0.89	0.84	0.04
${\dot{V}}_{O_{2}\mathrm{peak}}$ (L min^−1^)	2.84 ± 0.82	2.66 ± 0.99	0.74	0.20
${\dot{V}}_{O_{2}\mathrm{peak}}$ (mL·kg^−1^·min^−1^)	41.2 ± 10.5	39.0 ± 12.5	0.75	0.19
*W*_peak_ (W)	234 ± 113	225 ± 96	0.89	0.08

*Note*: ^a^Parametric values are presented as means ± SD, where data were analysed using independent *t*‐test and effect sizes were estimated using Cohen's *d*. Effect size: 0.2, small effect; 0.5, medium effect; 0.8, large effect. Non‐parametric values are presented as median (interquartile range), where data were analysed using Mann–Whitney *U*‐test and effect sizes were estimated using Rosenthal's *r*. Effect size: 0.2, weak effect; 0.4, moderate effect; 0.6, strong effect. Abbreviations: BMI, body mass index; BSA, body surface area; CON, control group; ES, effect size; ETI, Elexacaftor/Tezacaftor/Ivacaftor; F, female; FEF_25–75%_, forced mid‐expiratory flow; FEV_1_, forced expiratory volume in 1 s; F, female; FVC, forced vital capacity; M, male; pwCF, people with cystic fibrosis; SSA, mass‐specific surface area; ${\dot{V}}_{O_{2}\mathrm{peak}}$, peak oxygen uptake; *W*, peak power output.

For the e‐survey, a total of 30 pwCF (11 pwCF aged ≥16 years and 19 parents/guardians of young pwCF aged 6–15 years) completed the survey and were included in the analysis (*n *= 30). Inclusion criteria for pwCF aged ≥16 years included being under the care of a CF clinical care team, having a confirmed diagnosis of CF and fluency in English. Parents/guardians of pwCF aged 6–15 years answered on behalf of their child and were eligible if they were the primary caregiver of a child with CF and were fluent in English.

### 2.3 Experimental design

#### 2.3.1 Laboratory study

**FIGURE 1:**
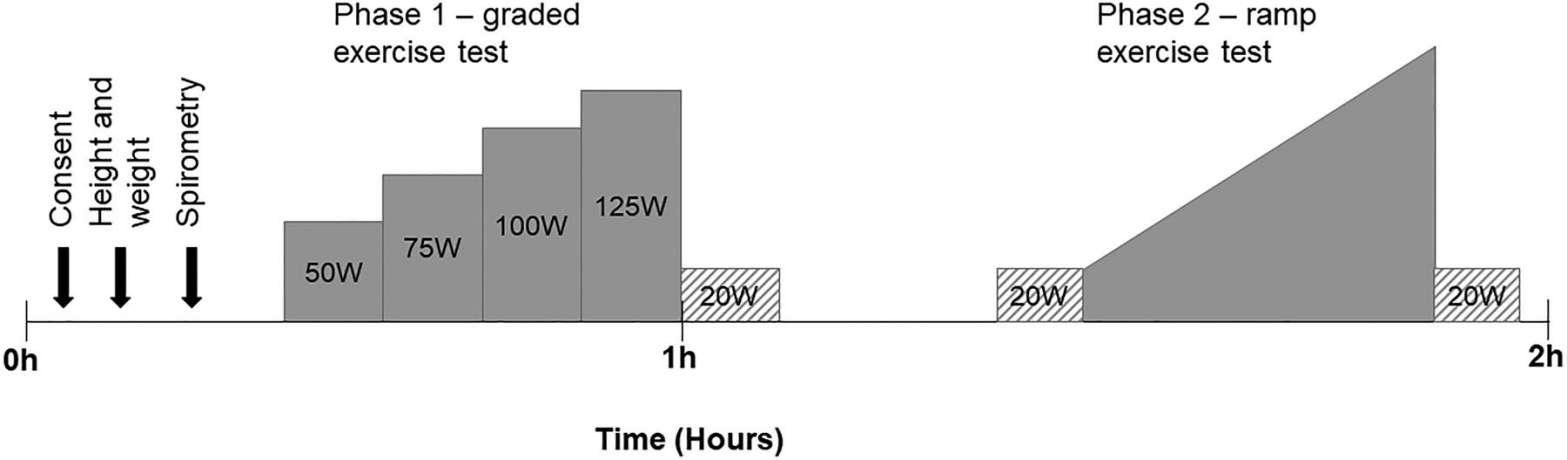
Schematic overview of visit 1 – preliminary assessments.

**FIGURE 2:**
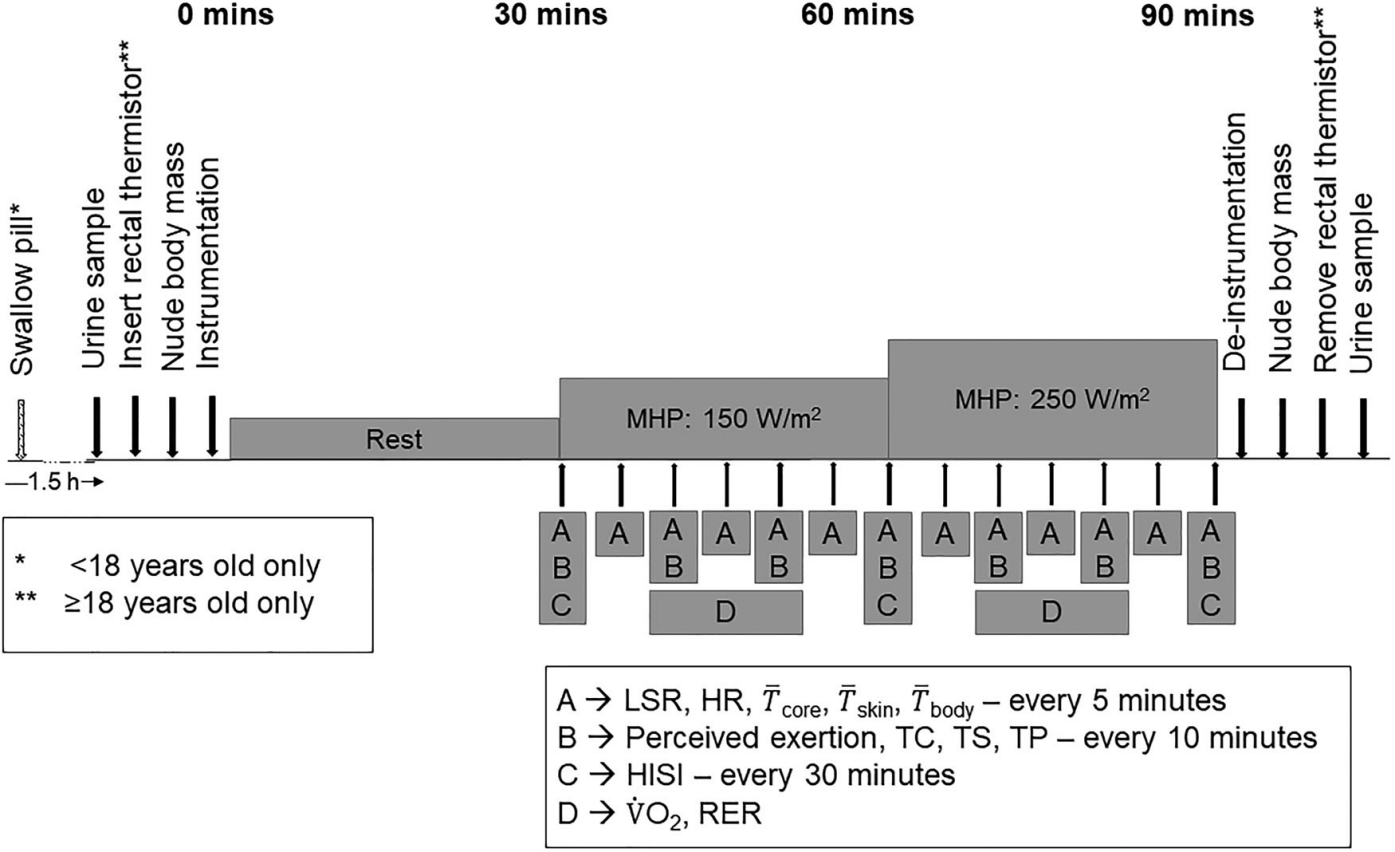
Schematic overview of the experimental session.

This single‐centre, cross‐sectional observational study involved two laboratory visits for each participant: (1) preliminary session (visit 1; Figure 1); (2) experimental session (visit 2; Figure 2), which were separated by a minimum of 24 h (5 ± 5 days) (Table 2). All visits were scheduled at the same time of day for each participant and their matched control. Participants arrived ≥ 2 h post‐prandial, having replicated their visit 1 diet for visit 2, and abstaining from alcohol, caffeine and intense physical activity for the preceding ≥ 24 h. During each visit, participants wore minimal clothing (underwear, shorts and trainers), with female participants also wearing a sports bra.

**TABLE 2:** Environmental, workload, metabolic heat production and hydration characteristics in response to a 1 h cycling exposure in the heat.

Variable	CON (*n *= 6)	pwCF (*n *= 6)	*P*	ES
WBGT (°C)	26.85 (25.84–27.04)	26.89 (26.68–27.91)	0.59	0.19
External workload (W)				
150 W·m^−2^	32 ± 16	31 ± 23	0.96	0.03
250 W·m^−2^	96 ± 36	92 ± 31	0.83	0.13
Absolute *H*_prod_ (W)				
150 W·m^−2^	296 ± 49	274 ± 67	0.53	0.38
250 W·m^−2^	495 ± 98	478 ± 93	0.77	0.18
Relative *H*_prod_ (W·m^−2^)				
150 W·m^−2^	164 ± 29	149 ± 13	0.28	0.66
250 W·m^−2^	271 ± 27	263 ± 21	0.60	0.32
Relative *H*_prod_ (W kg^−1^)				
150 W·m^−2^	4.5 ± 1.2	4.0 ± 0.3	0.38	0.53
250 W·m^−2^	6.8 (6.2–8.9)	6.7 (6.4–7.8)	0.94	0.05
Osmolality (mOsm kg^−1^)				
Pre‐exercise	495 ± 271	512 ± 222	0.91	0.07
Post‐exercise	521 ± 201	493 ± 229	0.82	0.13

*Note*: Parametric values are presented as means ± SD, where data were analysed using independent *t‐*test and effect sizes were estimated using Cohen's *d*. Effect size: 0.2, small effect; 0.5, medium effect; 0.8, large effect. Non‐parametric values are presented as median (interquartile range), where data were analysed using Mann–Whitney *U*‐test and effect sizes were estimated using Rosenthal's *r*.

Abbreviations: CON, control group; ES, effect size; *H*_prod_, metabolic heat production; pwCF, people with cystic fibrosis; WBGT, wet bulb globe temperature.

### 2.4 Visit 1: Preliminary assessments

During visit 1, stature was measured to the nearest 0.01 m using a stadiometer (Seca 213, UK), and baseline nude body mass to the nearest 0.01 kg using weighing scales (Industrial Electronic Weight Indicator, Model I10; Ohaus Corp., Parsippany, NJ, USA). BSA was subsequently calculated (DuBois, 1916). Pulmonary function was assessed via flow‐volume loop spirometry using a turbine flow‐meter system integrated within a metabolic cart (COSMED Ltd, Rome, Italy), following established guidelines (ATS, 2019). Participants subsequently completed a graded exercise test on an electromagnetically braked cycle ergometer (Lode Corival; Groningen, The Netherlands) to determine their MHP, the heat generated by the body as a by‐product of energy metabolism during physical activity or exercise, followed by an incremental extension phase to determine aerobic fitness (peak pulmonary oxygen uptake; ${\dot{V}}_{O_{2}\mathrm{peak}}$).

#### 2.4.1 Preliminary exercise test protocol

The preliminary exercise test to determine MHP and ${\dot{V}}_{O_{2}\mathrm{peak}}$ consisted of two distinct phases. Phase 1 involved four 5 minute stages at increasing external workloads (50 W, then +25 W per stage). Cadence was maintained at 70 rpm throughout. Participants then completed 5 minutes active recovery (20 W), followed by 15 minutes seated passive recovery.

Phase 2 comprised a minute‐by‐minute ramp incremental test to volitional exhaustion, designed to determine ${\dot{V}}_{O_{2}\mathrm{peak}}$ (see Figure 1). Following 3 minutes baseline cycling at 20 W, participants undertook the incremental ramp phase, with work rate increasing continuously (10–30 W·min^−1^), tailored to age, body mass and health status as has been suggested when exercise testing pwCF (Hulzebos et al., 2012). Participants maintained a cadence of 60–70 rpm throughout exercise until volitional exhaustion, defined as a sustained drop in cadence by ≥ 10 rpm consecutively for 5 s, despite strong verbal encouragement.

#### 2.4.2 Prescribing exercise intensity to elicit a fixed heat production during visit 2

Throughout the preliminary exercise testing, breath‐by‐breath changes in pulmonary gas exchange and ventilation were measured using a face mask (Hans Rudolph 7450 series V2 mask; Shawnee, KS, USA) connected to a turbine flowmeter system (COSMED). Data were displayed using an online gas analyser system (Quark CPET, COSMED). Pulmonary gas exchange and ventilation data from Phase 1 were interpolated to 1 second intervals and averaged every 1 minute. ${\dot{V}}_{O_{2}}$ and respiratory exchange ratio (RER) data from the final minute of each stage were used to calculate the rate of metabolic energy expenditure (*M*), in line with prior research (Jay & Cramer, 2016). Absolute MHP at each stage was then calculated as *M* minus external workload (*W*) and expressed relative to BSA (W·m^−2^). Results from each stage were then used to generate a regression equation, allowing accurate work rate prescription to elicit the target MHP.

### 2.5 Visit 2: Experimental assessments

Participants < 18 years old arrived at the laboratory 2 h prior to testing, to swallow an ingestible temperature capsule (e‐Celsius Performance; BodyCap, Hérouville‐Saint‐Clair, France), and returned 1.5 h later to continue procedures. Upon arrival, adult participants self‐inserted a rectal thermistor (Variohm Eurosensor; Towcester, UK), and all participants provided a baseline urine sample. Nude body mass was measured before participants dressed and were then instrumented in ambient conditions (∼22°C).

Participants then entered a climate‐controlled environmental chamber (35°C, 30% relative humidity) and rested for 30 minutes, to allow for stabilisation of physiological variables, before performing two consecutive 30 ‐minute cycling bouts (Figure 2). Exercise intensity was standardised to elicit a fixed rate of MHP relative to BSA, the recommended approach for comparison of sudomotor function (Cramer & Jay, 2014). Bout 1 targeted a MHP of 150 W·m^−2^, while the second bout targeted a MHP of 250 W·m^−2^ (Figure 2). Following exercise, participants exited the chamber, were de‐instrumented, nude body mass was reassessed to determine whole‐body sweat rate (WBSR; see details below), and a final urine sample was provided. Any fluid consumed or urine voided during the protocol was recorded and accounted for in the WBSR calculation.

### 2.6 Instrumentation

#### 2.6.1 Sweat response

After skin preparation, two sweat patches constructed of sterile Tegaderm™ (Tegaderm™ Dressings, 3M, St Paul, MN, USA), Parafilm^®^ (Bemis NA, Neenah, WI, USA), and sterile cotton wool were applied to the left scapular region. Post‐exercise, collected sweat samples were transferred to 1.5 mL Eppendorf^®^ tubes (Eppendorf UK, Stevenage, UK) and analysed using the Sweat‐Chek™ system (Wescor Biomedical Systems, Logan, UT, USA), to quantify [Na^+^Cl^−^].

Local sweat rate was measured using Q‐sweat capsules attached to the torso in right scapular region and on a limb, 2 cm below the right antecubital fossa (Q‐Sweat, WR TestWorks, interfaced with TestWorks software; WR Medical Electronics Co., Maplewood, MN, USA). Data were sampled every 0.25 seconds, averaged over 1 minute intervals, with the highest 1 minute average used as maximum sweat rate for each workload.

WBSR was determined using pre‐ and post‐exercise nude body mass, exercise duration, and fluid balance (accounting for fluid intake and urine output but not respiratory fluid losses), using:

(1) $$\begin{array}{ccc} \mathrm{WBSR} & = & \left[\left(\mathrm{massPre}\;\left(\mathrm{kg}\right)-\mathrm{massPost}\;\left(\mathrm{kg}\right)\right)+\mathrm{fluid}\;\mathrm{consumed}\;\left(\mathrm{kg}\right)\right.\\ & & \left.-\;\mathrm{urine}\;\mathrm{output}\;\left(\mathrm{kg}\right)\right]/\mathrm{exercise}\;\mathrm{duration}\;\left(h\right) \end{array}$$

#### 2.6.2 Core and skin temperature

For adults, core temperature (*T*_core_) was sampled every 1 second with values extracted at 1 minute intervals using a rectal thermistor (Variohm Eurosensor), self‐inserted 15 cm beyond the anal sphincter within a private cubicle, and then connected to a data logger (2020, Squirrel, Grant Instruments, Cambridge, UK). For paediatric participants, single use ingestible temperature pills (BodyCap e‐Celsius Performance), connected to a monitor (BodyCap eViewer performance monitor), were used. Data from these was subsequently extracted using the e‐Performance Manager software (v 1.1.0.13, BodyCap).

Skin temperature (*T*_skin_) was measured using skin thermistors (Grant Instruments) attached to four sites: midpoint of the right pectoralis major, the midpoint of the right triceps brachii lateral head, right rectus femoris and right gastrocnemius lateral head. These were attached to a data logger (2020, Squirrel). Mean *T*_skin_ was calculated every minute using the four skin temperatures (Ramanathan, 1964), and mean body temperature (*T*_body_) was calculated using a weighting of *T*_core_ and *T*_skin_ that is appropriate for exercise in a hot environment (Sawka et al., 2010).

#### 2.6.3 Cardiovascular response

Heart rate (HR) was recorded second‐by‐second (Polar Vantage V HR monitor; Polar Electro, Kempele, Finland) and averaged to 1 minute intervals. Peripheral skin blood flow (SkBF) was non‐invasively measured in the microvasculature of the middle finger pad of the left hand, and ventral surface of the left forearm throughout exercise, using a laser Doppler unit (Moor Instruments Ltd, Axminster, UK). Data were recorded in the PowerLab data acquisition system (ADInstruments, Bella Vista, Australia) and LabChart software (version 7, ADInstruments), sampling at 400 Hz. A biological zero was established before data collection, by occluding blood flow to the measurement site, with this value subtracted from the blood flow measurement during analysis.

#### 2.6.4 Perceptual responses

Perceptual measures of thermal comfort, thermal sensation, exertion and thirst were assessed at rest and every 10 minutes during exercise. Specifically, thermal comfort and thermal sensation were assed via a 20 cm visual analogue scale (0 cm, very comfortable, to 20 cm, very uncomfortable) and (0 cm, very hot, to 20 cm, very cold), respectively (Zhang, 2008). Ratings of perceived exertion were assessed using the Borg 6–20 scale (Borg, 1982), a 15‐point scale (6, no exertion, to 20, maximal exertion). Thirst perception was measured using a nine‐point scale (1, not thirsty, to 9, very, very thirsty) previously used in pwCF (Brown et al., 2011), and heat illness symptoms were assessed at rest and during the last minute of each 30 minute bout of exercise. Specifically, the heat‐illness symptom index (HISI) scale, a 10‐point index scale, was used to assess 13 symptoms rated on a scale from 0 (no symptoms) to 10 (had to stop exercise) (Coris et al., 2006).

#### 2.6.5 Hydration status

Participants were instructed to arrive in a euhydrated state (UOsm < 800 mOsm kg^−1^), by consuming 500 mL of water the night before and morning of testing (Sawka et al., 2009). Urine osmolality was assessed from a fresh mid‐flow urine sample obtained upon arrival, using an Osmocheck Urine Analysis Unit (Osmocheck, Vitech Scientific, Horsham, UK). Participants who were dehydrated (UOsm > 800 mOsm kg^−1^) (Sawka et al., 2009) were provided with a 300 mL bolus of water, with urine osmolality re‐assessed before entering the environmental chamber.

#### 2.6.6 Metabolic heat production

Breath‐by‐breath changes in pulmonary gas exchange and ventilation were collected during the middle 10 minute period during each 30 minute bout of cycling to calculate the calculate the rate of metabolic energy expenditure and, subsequently, MHP, to verify that the target MHP (150 and 250 W·m^−2^, respectively) had been achieved.

### 2.7 eSurvey study

Separate surveys were designed for pwCF aged ≥ 16 years and parents/guardians of pwCF aged 6–15 years. Surveys were disseminated globally via social media platforms managed by members of the research team, the CF Warriors charity, and the CF Australia charity. Both surveys contained 41 questions, across four sections: (1) demographic information; (2) the International Physical Activity Questionnaire (IPAQ) (Craig et al., 2003); (3) experiences of sweating, hydration, and heat‐related illness; and (4) dietary salt and electrolyte supplementation intake.

### 2.8 Data analysis

In the laboratory study, three participants were unable to complete the full 30 minute bout at 250 W·m^−2^ due to volitional exhaustion (CF: *n *= 1) or reaching their predicted HR maximum requiring exercise cessation (CF: *n *= 1; CON: *n *= 1), in accordance with predefined safety criteria. Data beyond the termination point were excluded from the analysis, along with corresponding data from their matched pairs. Additionally, due to technical issues, HR data for one healthy control, and local back sweat rate for one healthy control participant, were not obtained; the corresponding data from their matched pairs were also removed for these measures prior to analysis.

In the e‐survey, *n *= 30 completed the survey and were included in the analysis. Data were summarised as counts (*n*) and frequencies (%), with open text responses providing further detail where appropriate.

#### 2.8.1 Statistical analyses

Statistical analyses were conducted using IBM SPSS Statistics software (version 27.0; IBM Corp., Armonk, NY, USA). Normality was assessed with the Shapiro–Wilk test. Normally distributed data are presented as means ± standard deviation (SD) and were analysed using Student's independent samples *t*‐test. Effect size (ES) was estimated using Cohen's *d*, classified as small (*d *= 0.2), medium (*d *= 0.5) or large (*d *= 0.8). Data that were not normally distributed data are presented as medians and interquartile ranges (25^th^ and 75^th^ percentiles) and were analysed using the Mann–Whitney *U*‐test. *ES* was estimated using Rosenthal's *r*, classified as weak (*r *= 0.2), moderate (*r *= 0.4) or strong (*r *= 0.6). Statistical significance was set at *P *< 0.05.

## 3 RESULTS

### 3.1 Laboratory study

#### 3.1.1 Participant characteristics

Participant characteristics are presented in Table 1. There were no significant differences between groups in age, stature, body mass, BSA, body mass index, lung function, ${\dot{V}}_{O_{2}\mathrm{peak}}$ or peak power output (*W*_peak_) (all *P* > 0.05), all of which had a small ES.

#### 3.1.2 Thermoregulatory responses to exercise heat stress

Ambient environmental conditions, MHP, workload, and pre‐ and post‐exercise hydration status during visit 2 (experimental session) were not different between groups (Table 2).

#### 3.1.3 Sweat responses

**FIGURE 3:**
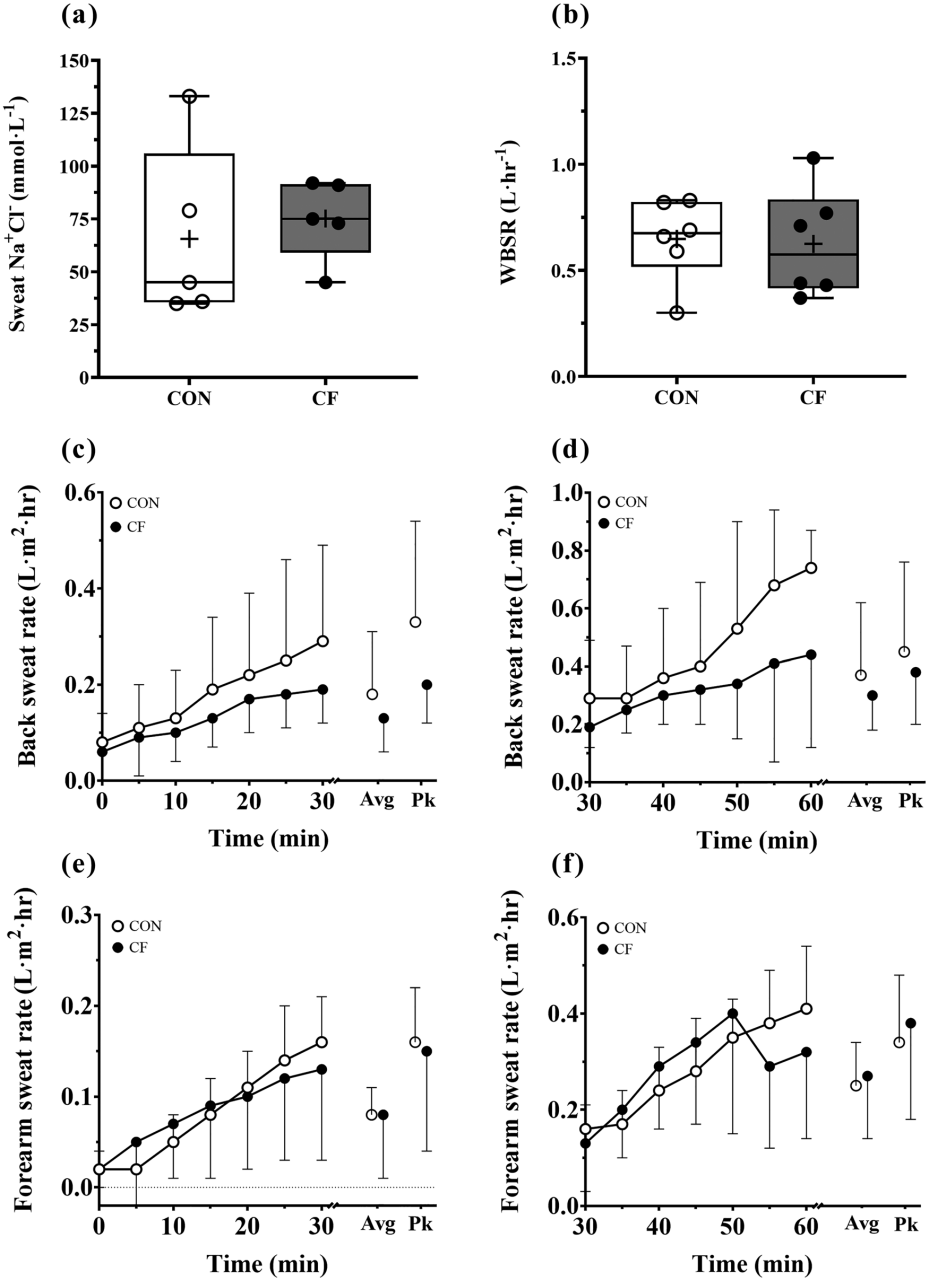
Parameters for sweat [Na^+^Cl^−^], whole‐body sweat rate and local sweat rate on the back and forearm in people with cystic fibrosis compared with healthy age‐, sex‐, height‐ and weight‐matched control participants. (a, b) Data are presented as the median (mid‐line), mean (cross), 25^th^ and 75^th^ interquartile ranges (boxes) and the highest and lowest values (whiskers) with individual data points for sweat [Na^+^Cl^−^] (a) and whole‐body sweat rate (b) in control (white) and pwCF (black). (c–f) Data are presented as mean and standard deviation for 5‐minute intervals, average and peak, local back sweat rate during cycling exercise at intensity 1 (150 W·m^−2^) (c), and intensity 2 (250 W·m^−2^) (d), and local forearm sweat rate during cycling exercise at intensity 1 (150 W·m^−2^) (e), and intensity 2 (250 W·m^−2^) (f). Avg, average; CON, healthy control; Pk, peak; pwCF, people with cystic fibrosis.

Sweat [Na^+^Cl^−^] was not significantly different between pwCF and healthy controls (CON: 45 (36–106) mmol·L^−1^; pwCF: 75 (59–92) mmol·L^−1^, *P *= 0.45, *z *= −0.84, *r *= 0.26; Figure 3a). Local sweat rate on the back and forearm at MHP of 150 W·m^−2^ and 250 W·m^−2^ are presented in Figure 3. Average back sweat rate (CON: 0.13 (0.09–0.31) L m^2^ h^−1^; pwCF: 0.10 (0.08–0.20) L m^2^ h^−1^, *P *= 0.65, *z *= −0.52, *r *= 0.17) and peak back sweat rate (CON: 0.22 (0.16–0.55) L m^2^ h^−1^; pwCF: 0.18 (0.15–0.28) L m^2^ h^−1^, *P *= 0.55, *z *= −0.73, *r *= 0.23) during MHP of 150 W·m^−2^, as well as average back sweat rate (CON: 0.25 (0.16–0.64) L m^2^ h^−1^; pwCF: 0.28 (0.21–0.41) L m^2^ h^−1^, *P *= 1.00, *z *= −0.00, *r *= 0.00) and peak back sweat rate (CON: 0.29 (0.20–0.79) L m^2^ h^−1^; pwCF: 0.30 (0.26–0.55) L m^2^ h^−1^, *P *= 0.81, *z *= −0.32, *r *= 0.10) at MHP of 250 W·m^−2^, were not different between groups (Figure 3c,d).

Average forearm sweat rate (CON: 0.08 ± 0.03 L m^2^ h^−1^; pwCF: 0.09 ± 0.07 L m^2^ h^−1^, *P *= 0.92, *t *= 0.11, *d *= 0.06) and peak forearm sweat rate (CON: 0.16 ± 0.06 L m^2^ h^−1^; pwCF: 0.15 ± 0.11 L m^2^ h^−1^, *P *= 0.80, *t *= −0.26, *d *= 0.15) at MHP of 150 W·m^−2^, as well as average forearm sweat rate (CON: 0.25 ± 0.09 L m^2^ h^−1^; pwCF: 0.28 ± 0.13 L m^2^ h^−1^, *P *= 0.71, *t *= 0.38, *d *= 0.22) and peak forearm sweat rate (CON: 0.34 ± 0.14 L m^2^ h^−1^; pwCF: 0.38 ± 0.20 L m^2^ h^−1^, *P *= 0.66, *t *= 0.45, *d *= 0.26) at MHP of 250 W·m^−2^, were not significantly different between groups (Figure 3e,f). Additionally, there were no significant difference in WBSR (CON: 0.65 ± 0.19 L h^−1^; pwCF: 0.63 ± 0.26 L h^−1^, *P *= 0.86, *t *= −0.18, *d *= 0.10; Figure 3b).

#### 3.1.4 Core and mean skin temperatures

Baseline $\bar{T}$_core_ was not significantly different between pwCF and CON (*P *> 0.05). Similarly, peak $\bar{T}$_core_ and ∆$\bar{T}$_core_ during cycling at MHP of 150 and 250 W·m^−2^ were not different between groups (all *P *> 0.05; Table 3). Baseline $\bar{T}$_skin_ was also not significantly different between groups (*P *> 0.05). No significant differences were observed in peak $\bar{T}$_skin_ and ∆$\bar{T}$_skin_ at MHP of 150 W·m^−2^ and 250 W·m^−2^ (all *P *> 0.05; Table 3). Baseline $\bar{T}$_body_ showed no differences between groups (*P *> 0.05), and no significant between group differences were observed in peak $\bar{T}$_body_ or ∆$\bar{T}$_body_ at MHP of 150 and 250 W·m^−2^ (all *P *> 0.05; Table 3).

**TABLE 3:** Baseline, peak and change in $\bar{T}$_core_, $\bar{T}$_skin_ and $\bar{T}$_body_ during exercise experimental exposures to the heat in the temperature‐controlled environmental chamber.

Variable	CON (*n *= 6)	pwCF (*n *= 6)	*P*	ES
Baseline $\bar{T}$_core_	37.01 ± 0.41	37.07 ± 0.27	0.78	0.17
Peak $\bar{T}$_core_				
150 W·m^−2^	37.35 ± 0.20	37.27 ± 0.27	0.58	0.33
250 W·m^−2^	37.72 ± 0.13	37.73 ± 0.35	0.97	0.03
**∆** $\bar{T}$_core_				
150 W·m^−2^	0.34 ± 0.35	0.20 ± 0.14	0.40	0.50
250 W·m^−2^	0.50 ± 0.16	0.51 ± 0.80	0.98	0.02
Baseline $\bar{T}$_skin_	35.04 ± 0.31	35.15 ± 0.41	0.62	0.30
Peak $\bar{T}$_skin_				
150 W·m^−2^	35.87 (35.78 – 36.30)	36.05 (35.7–36.42)	0.62	0.16
250 W·m^−2^	36.16 ± 0.26	36.46 ± 0.33	0.11	**1.01**
**∆** $\bar{T}$_skin_				
150 W·m^−2^	1.02 (0.84 – 1.18)	0.67 (0.62–1.19)	0.31	0.32
250 W·m^−2^	0.41 ± 0.16	0.55 ± 0.34	0.40	0.51
Baseline $\bar{T}$_body_	36.81 ± 0.37	36.88 ± 0.26	0.74	0.20
Peak $\bar{T}$_body_				
150 W·m^−2^	37.17 ± 0.17	37.13 ± 0.25	0.73	0.21
250 W·m^−2^	37.56 ± 0.12	37.59 ± 0.35	0.83	0.13
**∆** $\bar{T}$_body_				
150 W·m^−2^	0.36 ± 0.33	0.25 ± 0.16	0.49	0.42
250 W·m^−2^	0.49 ± 0.16	0.50 ± 0.19	0.91	0.07

*Note*: Parametric values are presented as means ± SD where data were analysed using independent *t*‐test and effect sizes were estimated using Cohen's *d*. Effect size: 0.2, small effect; 0.5, medium effect; 0.8, large effect. Bold text denotes a large effect size. Non‐parametric values are presented as median (interquartile range), where data were analysed using Mann–Whitney *U*‐test and effect sizes were estimated using Rosenthal's *r*.

Abbreviations: CON, control group; ES, effect size; pwCF, people with cystic fibrosis; $\bar{T}$_body_, mean body temperature; $\bar{T}$_core_, core temperature; $\bar{T}$_skin_, mean skin temperature.

#### 3.1.5 Cardiovascular response

There were no significant differences between pwCF and CON in baseline HR, or average and peak HR, during cycling at MHP of 150 and 250 W·m^−2^ (all *P *> 0.05; Table 4). Similarly, peripheral SkBF at the middle finger pad and forearm showed no significant differences between groups at baseline, and average and peak SkBF during both exercise intensities remained comparable (all *P *> 0.05; Table 4).

**TABLE 4:** Baseline, average and peak heart rate and skin blood flow measurements during exercise experimental exposures to the heat in a temperature‐controlled environmental chamber.

Variable	CON (*n *= 6)	pwCF (*n *= 6)	*P*	ES
HR (beats·min^−1^)				
Baseline	83 (75–100)	85 (78–108)	0.79	0.10
Average 150 W·m^−2^	92 (86–117)	99 (85–121)	1.00	0.03
Peak 150 W·m^−2^	97 (94–124)	105 (91–151)	1.00	0.03
Average 250 W·m^−2^	120 (113–161)	133 (115–164)	0.84	0.10
Peak 250 W·m^−2^	130 (125–171)	139 (123–176)	0.84	0.10
Finger SkBF (LDU)				
Baseline	237.0 ± 73.0	255.9 ± 80.2	0.68	0.25
Average 150 W·m^−2^	249.0 ± 45.8	276.3 ± 47.0	0.33	0.59
Peak 150 W·m^−2^	332.1 ± 54.1	333.2 ± 545.1	0.97	0.02
Average 250 W·m^−2^	300.0 ± 35.7	291.8 ± 70.6	0.81	0.15
Peak 250 W·m^−2^	366.6 ± 39.5	326.7 ± 73.0	0.27	0.68
Forearm SkBF (LDU)				
Baseline	44.6 ± 21.2	38.6 ± 23.6	0.66	0.27
Average 150 W·m^−2^	81.4 ± 31.8	84.5 ± 13.9	0.85	0.11
Peak 150 W·m^−2^	138.0 ± 57.1	138.0 ± 47.0	0.98	0.01
Average 250 W·m^−2^	165.0 ± 64.9	175.2 ± 29.3	0.73	0.20
Peak 250 W·m^−2^	212.5 ± 91.8	231.2 ± 49.2	0.67	0.25

*Note*: Parametric values are presented as means ± SD, where data were analysed using independent *t*‐test and effect sizes were estimated using Cohen's *d*. Effect size: 0.2, small effect; 0.5, medium effect; 0.8, large effect. Non‐parametric values are presented as median (interquartile range), where data were analysed using Mann–Whitney *U*‐test and effect sizes were estimated using Rosenthal's *r*.

Abbreviations: CON, control group; ES, effect size; HR, heart rate; LDU, laser Doppler units; pwCF, people with cystic fibrosis; SkBF, skin blood flow.

#### 3.1.6 Perceptual response

Thirst perception was significantly greater in pwCF than CON at baseline (*P = *0.045, *r *= 0.67) and at the end of 150 W·m^−2^ (*P *= 0.01, *r *= 0.78). However, thirst perception at the end of 250 W·m^−2^ (*P *= 0.15, *r *= 0.53) was not significantly different between groups (Table 5). Perceived exertion, thermal comfort, thermal sensation and HISI at baseline and the end of 150 and 250 W·m^−2^ stages were not significantly different between groups (all *P *> 0.05; Table 5).

**TABLE 5:** Perceptual responses during exercise experimental exposures to the heat in a temperature‐controlled environmental chamber at rest and in response to two target metabolic heat productions (150 W·m^−2^ and 250 W·m^−2^).

Variable	CON	pwCF	*P*	ES
Perceived exertion (a.u.)				
Baseline	6 (6–6)	6 (6–6)	1.00	0.29
150 W·m^−2^	8 ± 2	8 ± 1	1.00	0.00
250 W·m^−2^	13 ± 4	13 ± 4	1.00	0.00
Thermal comfort (cm)				
Baseline	15.8 ± 2.7	14.9 ± 3.3	0.61	0.30
150 W·m^−2^	13.8 ± 3.4	13.4 ± 4.0	0.86	0.11
250 W·m^−2^	11.7 ± 4.7	10.3 ± 6.3	0.67	0.25
Thermal sensation (cm)				
Baseline	14.1 ± 2.5	13.4 ± 1.3	0.59	0.33
150 W·m^−2^	15.5 ± 2.0	14.5 ± 1.8	0.43	0.47
250 W·m^−2^	17.35 (13.6–18.08)	16.8 (12–17.35)	0.31	0.32
Thirst perception (a.u.)				
Baseline	1 (1–1)	3 (1–4)	< 0.05^*^	0.67
150 W·m^−2^	1 (1–2)	3 (3–4)	0.01^*^	0.79
250 W·m^−2^	3 (3–5)	5 (5–5)	0.15	0.53
HISI (a.u.)				
Baseline	1 ± 2	3 ± 3	0.17	0.86
150 W·m^−2^	4 ± 3	6 ± 3	0.32	0.60
250 W·m^−2^	13 ± 10	18 ± 13	0.44	0.47

*Note*: Parametric values are presented as means ± SD, where data were analysed using independent *t*‐test and effect sizes were estimated using Cohen's *d*. Effect size: 0.2, small effect; 0.5, medium effect; 0.8, large effect. Non‐parametric values are presented as median (interquartile range), where data were analysed using Mann–Whitney *U*‐test and effect sizes were estimated using Rosenthal's *r*. ^*^Statistical difference (*P *< 0.05).

Abbreviations: CON, control group; ES, effect size; HR, heart rate; pwCF, people with cystic fibrosis; HISI, heat‐illness symptom index.

### 3.2 e‐Survey study

Detailed survey data are available in the Appendix Tables A1, A2, A3, A4, A5, A6, A7, A8. Primary findings from our survey revealed that 40% (*n* = 12) of pwCF reported that they perceived differences in the amount they sweat since starting CFTR modulator therapy, with 43.3% (*n* = 13) perceiving a difference with how salty their sweat is. Fatigue and thirst were the most commonly reported heat‐related illness symptoms, with cramps, nausea, dizziness, muscle weakness, heat sensations on head or neck, feeling light‐headed and a dry mouth also prevalent in the heat (Table A4). Moreover, 40% (*n* = 12) of those surveyed reported changes in dehydration or heat‐related illness symptoms since starting CFTR modulator therapy, for a variety of reasons (Table A5). Also, 36.7% of respondents reported taking salt supplements, with 33.3% adding extra salt to their food, and 43.3% consuming electrolyte drinks during rest, exercise or in hot environments. Of the respondents, 63% reported that they have not adjusted their salt intake since commencing CFTR modulator therapy.

## 4 DISCUSSION

This study is the first to adopt a mixed‐methods, multi‐study approach to investigate thermophysiological and perceptual responses to laboratory‐controlled exercise heat‐stress in pwCF receiving CFTR modulator therapy, alongside exploratory pilot survey insights into real‐world sweating perceptions, as well as hydration and salt replacement strategies. We provide the first tightly controlled laboratory data, providing comprehensive and contemporary characterisation of thermoregulation, sweating and heat‐illness vulnerability in pwCF treated with ETI, as well as self‐reported experiences. Across both studies, four principal novel findings emerged: during prolonged exercise in the heat at a fixed rate of metabolic heat production, (1) pwCF on ETI demonstrated thermoregulatory and perceptual responses that were not different from healthy matched controls, (2) with the exception of greater thirst perception in pwCF both at rest and during exercise at the lowest metabolic heat production (150 W·m^−2^); (3) exercise sweat [Na^+^Cl^−^] did not differ between groups, challenging long‐standing characteristic excessive salt loss in pwCF prior to CFTR modulator therapies; and (4) many pwCF on ETI reported clear perceptual changes in sweating, sweat saltiness, thirst and heat‐illness symptoms in free‐living conditions since commencing treatment. Together, these findings provide the first evidence that ETI substantially alters heat‐ and hydration‐related perceptions in people with CF and that their thermophysiolgical responses to exercise heat stress do not differ from healthy matched controls, with direct implications for hydration and salt‐supplementation guidance.

### 4.1 Laboratory findings: normalisation of thermoregulatory and sweating responses during exercise heat‐stress

Our laboratory study provides the first controlled physiological evidence that pwCF on ETI exhibit thermoregulatory and, for the most part, perceptual responses that are not different from healthy matched controls during prolonged cycling in the heat (35°C, 30% RH) at a fixed metabolic heat production (150 W·m^−^^2^). No group differences were observed for sweat [Na^+^Cl^−^], whole‐body or local sweat rates, core or skin temperatures, mean body temperature, heart rate, or cutaneous skin blood flow. These findings contrast sharply with the limited existing research before the availability of CFTR modulator therapy (Brown et al., 2011; Orenstein et al., 1983).

In the only prior study to have examined exercise sweat [Na^+^Cl^−^] in pwCF, significantly elevated exercise sweat [Na^+^Cl^−^] in pwCF during 70 minutes of cycling at 50% ${\dot{V}}_{O_{2}\mathrm{peak}}$ in the heat (37–38°C dry bulb, 24–29°C wet bulb) were reported. While methodological differences and technological advancements may partly explain this contrast, the most plausible explanation is the transformative effects of ETI on sweat gland function (McKone et al., 2014; Ramsey et al., 2011; Taylor‐Cousar et al., 2017), specifically, reductions in resting sweat [Cl^−^] following ETI treatment (Zemanick *et al.*, 2021). CFTR proteins, functionally active in eccrine sweat glands, are involved in β‐adrenergic (cAMP) sweat secretion in the secretory coil and facilitate the absorption of Cl^−^ and regulation of Na^+^ absorption via the epithelial Na^+^ channel in the re‐absorptive duct (Saint‐Criq & Gray, 2017). Dysfunctional CFTR proteins impair Na^+^Cl^−^ re‐absorption (Saint‐Criq & Gray, 2017) leading, historically, to 3–5 times higher salt loss than in healthy individuals (Emrich et al., 1968). CTFR modulators have been shown to significantly reduce sweat [Cl^−^] at rest (Heijerman et al., 2019; Middleton et al., 2019), with a reduction of 60.9 mmol·L^−1^ following 24 weeks of ETI (Zemanick et al., 2021).

This study is the first of its kind to investigate thermophysiological and perceptual responses to exercise in the heat in pwCF on CFTR modulator therapy; revealing comparable $\bar{T}$_core_, $\bar{T}$_skin_, $\bar{T}$_body_, WBSR, local back sweat rate, local forearm sweat rate, HR, or SkBF to healthy matched controls. Pre‐CFTR modulator data also revealed no differences in peak $\bar{T}$_core_ or HR during 70 minutes of cycling at 50% ${\dot{V}}_{O_{2}\mathrm{peak}}$ in the heat. However, several methodological limitations may have influenced these findings. Matching for age, sex, height and weight, as in the present study, is crucial and a major methodological advancement, given their influence on thermoregulatory responses (Gagnon et al., 2008; Jay & Cramer, 2016). Additionally, prescribing workload based on % ${\dot{V}}_{O_{2}\mathrm{peak}}$ can lead to systematic differences in MHP and variations in $\bar{T}$_core_ response (Cramer & Jay, 2015). Beyond these methodological improvements, the present study employed direct measures of thermoregulatory function, including core and skin temperature, sweat rate and perceptual responses, which provide much greater insight into thermoregulation in pwCF.

### 4.2 Free‐living perceptions of sweating, saltiness, dehydration and heat‐related illness in the ETI era

The e‐survey revealed that 40% (*n* = 12) of pwCF reported perceived differences in the amount they sweat since starting CFTR modulator therapy, and 43.3% (*n* = 13) perceived differences with how salty their sweat is, with many feeling that they get less sweaty and lose less salt. These perceptions are important as they may indicate CFTR modulator‐related alterations in sweat gland function and electrolyte composition. In particular, a reduction in sweat [Na^+^Cl^−^], if not accompanied by a corresponding adjustment in salt supplementation, may increase the risk of excessive salt intake. Importantly, 40% of respondents also reported changes in dehydration or heat‐related illness symptoms, with many indicating improvements (e.g., fewer cramps, headaches and less fatigue). These qualitative insights, coupled with perceived changes in sweat composition, suggest that ETI may modify heat‐related symptomatology and hydration, potentially through restored electrolyte handling and altered fluid‐regulatory signalling. Despite this, many pwCF (63%) have reportedly not adjusted their salt intake since commencing CFTR modulator therapy, suggesting that further research is needed to determine whether current supplementation practices remain appropriate following modulator therapy.

### 4.3 Heightened thirst: potential restoration of fluid‐regulatory mechanisms

A novel observation in the laboratory study was also the heightened thirst perception in pwCF at baseline and during cycling in the heat. Historically, excessive sweat salt loss and an attenuated body‐fluid hyperosmolality has blunted the hyperosmotic trigger for thirst, increasing the risk of voluntary dehydration in pwCF (Bar‐Or et al., 1992; Brown et al., 2011). Before CFTR modulator therapy, this contributed to greater dehydration risk both during (Bar‐Or et al., 1992) and following (Brown et al., 2011) exercise in the heat. The reduction in salt loss with ETI may restore normal fluid regulatory mechanisms, enhancing thirst drive and promoting better hydration routines in pwCF. Importantly, however, urine osmolality did not differ between groups, indicating that heightened thirst occurred in the absence of measurable differences in hydration status. This dissociation suggests that perceptual changes may reflect altered fluid‐regulatory sensitivity rather than systemic dehydration, and warrants further large‐scale investigation, including the measurement of plasma osmolality. Survey responses further support this, with some pwCF noting changes in water consumption habits since starting CFTR modulator therapy, including:

‘I feel as though I need to drink more water because I feel ‘drier’ in general on the Trikafta’ (Elexacaftor/Tezacaftor/Ivacaftor) [39 years old, Australia].

‘[I'm] always thirsty. Hard to quench. Sliced cucumbers help with quench’ (61 years old, Australia)

### 4.4 Heat dissipation mechanisms: Preserved cutaneous vasodilation in pwCF on ETI

Effectively regulating body temperature during exercise in the heat requires balancing MHP with total heat loss (Baker, 2019), primarily through cutaneous vasodilation, which increases skin blood flow and facilitates heat transfer from the body core to the skin (Jay & Cramer, 2016). Although relevant data were previously lacking, we hypothesised that CFTR dysfunction in pwCF may impair heat dissipation, as previous studies reported reduced micro‐ and macrovascular function in pwCF prior to CFTR modulator therapy (Poore et al., 2013; Rodriguez‐Miguelez et al., 2016), and we have recently demonstrated some persistent vascular abnormalities in those treated with ETI (Clayton et al., 2025). Despite these concerns, the present findings demonstrate that SkBF during exercise heat‐stress at fixed rates of MHP does not differ between pwCF established on ETI and healthy matched controls. This suggests that cutaneous vascular responses to heat stress may remain intact in individuals receiving ETI, although further research is warranted, particularly given methodological criticisms of laser Doppler flowmetry, which provides only relative perfusion estimates and is sensitive to probe placement and reproducibility issues (Rajan et al., 2009).

Despite the historical association between CF and heat illness (Kessler & Andersen, 1951), as well as salt losses (di Sant'Agnese et al., 1953), research into sweating, thermoregulation, and perceptual responses in pwCF in the context of physical activity and exercise has been scarce. As physical activity and structured exercise become increasingly central to the management of an ageing CF population, the need to understand heat‐stress responses to the changing climate has never been greater. This urgency is amplified by rising global temperatures (NOAA National Centers for Environmental Information, 2023), CFTR modulators reshaping disease pathophysiology for those with access, and growing concerns regarding cardiovascular manifestations in this population. The present findings highlight the need for further research to establish whether hydration and salt supplementation recommendations remain appropriate following CFTR modulator therapy, particularly given the potential health implications of both inadequate and excessive salt intake. Since access to and tolerance of modulators is not universal, there remains a pressing need for inclusive research and tailored clinical guidance, and future studies should also include people both receiving and not receiving modulator therapy to inform evidence‐based guidance.

### 4.5 Implications for clinical practice and guideline revision

Historically, elevated sweat [Na^+^Cl^−^] in pwCF has driven salt supplementation guidelines; however, current recommendations remain inconsistent and non‐specific with regard to exercise, particularly exercise in the heat and, indeed, no formal exercise‐specific hydration guidelines currently exist (Wilschanski et al., 2024). The introduction of CFTR modulator therapies complicates this further. Our findings in these studies suggest that hydration and salt supplementation guidelines, largely unchanged since the pre‐modulator era, require urgent re‐evaluation. Given heterogeneity in modulator access and tolerability, future guidance must be individualised and reflect the diverging physiological profiles of pwCF on and off CFTR modulators.

Our exploratory pilot e‐survey findings revealed that, despite being on CFTR modulator therapy, 36.7% of respondents take salt supplements, 33.3% add extra salt to their food, and 43.3% consume electrolyte drinks during rest, exercise or in hot environments. Meanwhile, many (63%) have not adjusted their intake since commencing CFTR modulator therapy. Given the observation that ETI may restore sweat [Na^+^Cl^−^] to levels comparable to healthy controls, the routine use of salt supplementation may no longer be necessary for individuals on ETI. Indeed, excessive salt intake may have negative health consequences, such as increased blood pressure or impaired renal function (Malta et al., 2018). Assessment of urinary sodium excretion could provide further insight into sodium handling, particularly where sweat differences are absent.

This aligns with a recent study (Berg et al., 2025) that comprehensively assessed the impact of ETI on electrolyte and volume homeostasis in pwCF. Treatment of 50 pwCF with ETI was shown to increase Na^+^ and volume retention, reflected by rises in blood pressure, body weight, plasma Na^+^, urine Na^+^/K^+^ ratio, and enhanced diuretic and natriuretic responses to volume and NaHCO_3_ loading, while heart rate, plasma aldosterone and bicarbonate decreased. These changes are consistent with a largely normalised sweat electrolyte concentration, indicating that the salt‑wasting phenotype and volume contraction characteristic of CF are significantly improved with ETI treatment. Importantly, this has clinical implications, as routine Na^+^Cl^−^ replacement therapy may need to be reconsidered to avoid excessive supplementation. Our study extends these observations by examining exercise and heat‑stress responses, further underscoring the need to update hydration and salt supplementation guidelines in the context of CFTR modulator therapy.

Together, these findings suggest that excess supplementation may simply be filtered and excreted at the renal level, although further exercise heat‐stress research with measurement is needed to confirm this, underscoring the need to reassess salt‑replacement strategies in pwCF receiving ETI. As CF population now ages and develops common comorbidities such as cardiovascular disease (Frost et al., 2023), maintaining an appropriate dietary salt intake becomes increasingly critical, reinforcing, and the present findings provide some preliminary evidence to inform future research focused on understanding hydration and salt supplementation in CF, which many inform clinical recommendations in the future.

### 4.6 Strengths, limitation and future directions

Major strengths of this work include the matched control design (age, sex, height, weight), fixed‐MHP exercise prescription, and integration of mechanistic laboratory assessments with ecologically valid free‐living data – an approach not previously applied in CF research. However, several limitations must be acknowledged. These include the single‐centre design, modest sample size, high inter‐individual variability in sweat [Na^+^Cl^−^], and the inability to obtain pre‐ and post‐ETI data or a comparison group not receiving ETI. Given the widespread availability of ETI in the UK, this was not feasible; however, future studies in regions where access to ETI remains variable may help determine whether thermoregulatory responses differ between individuals receiving and not receiving modulator therapy. The pilot survey was exploratory, not powered for group comparisons, and included a small, mixed respondent sample; therefore, findings should be interpreted with caution.

Additionally, the absence of serum and urine sodium measurements limits interpretation of systemic sodium handling, as such data could have provided valuable insight into whether supplementation was retained or excreted at the renal level. As the first in the world proof‐of‐concept study in pwCF, these findings provide valuable insight, but larger studies are needed to confirm restored fluid‐regulatory mechanisms, evaluate heat‐illness susceptibility in the ageing CF population who appear to carry a growing cardiovascular disease risk, and refine hydration and heat‐mitigation guidance during physical activity, particularly in hot environments.

### 4.7 Conclusion

These mixed‐methods, multi‐study findings provide the first comprehensive evaluation of heat‐related physiology and free‐living perceptions and experiences in pwCF treated with ETI. Our findings provide no evidence of abnormal sweating and thermoregulatory responses to exercise at a fixed rate of MHP under heat stress in pwCF on ETI when compared to healthy, matched controls. Additionally, free‐living experiences suggest changes in sweating patterns and heat illness symptoms post‐CFTR modulator therapy. These results challenge longstanding assumptions regarding excessive salt loss in CF and underscore the need to re‐evaluate hydration and salt supplementation guidelines for pwCF, with consideration of current CFTR modulator treatment regimens. This work addresses an urgent evidence gap with direct implications for clinical practice, patient safety and heat‐health resilience in a warming climate.

## AUTHOR CONTRIBUTIONS

Lauren J. Clayton: conceptualisation, methodology, data curation, investigation, writing ‐original draft, writing—review and editing, project administration, resources, formal analysis. Jo Corbett: supervision, conceptualisation, methodology, funding acquisition, resources, project administration, writing—original draft, writing—review and editing, data curation, investigation. Anthony I. Shepherd: supervision, conceptualisation, methodology, funding acquisition, resources, project administration, writing—original draft, writing—review and editing, data curation, investigation. Heather Massey: supervision, conceptualisation, methodology, funding acquisition, resources, project administration, writing—original draft, writing—review and editing, data curation, investigation. Don S. Urquhart:  conceptualisation, methodology, writing—original draft, writing—review and editing, data curation. Neil Maxwell:  conceptualisation, methodology, funding acquisition, resources, writing—original draft, writing—review and editing, data curation. Gary Connett:  conceptualisation, methodology, writing—original draft, writing—review and editing, data curation. Mark Allenby:  conceptualisation, methodology, writing—original draft, writing—review and editing, data curation. Julian Legg:  conceptualisation, methodology, writing—original draft, writing—review and editing, data curation. Thomas Daniels:  conceptualisation, methodology, writing—original draft, writing—review and editing, data curation. Zoe L. Saynor: supervision, conceptualisation, methodology, funding acquisition, resources, project administration, investigation, writing—original draft, writing—review and editing, data curation. All authors have read and approved the final version of this manuscript and agree to be accountable for all aspects of the work in ensuring that questions related to the accuracy or integrity of any part of the work are appropriately investigated and resolved. All persons designated as authors qualify for authorship, and all those who qualify for authorship are listed.

## CONFLICT OF INTEREST

G.C. and D.S.U. have both served as principal investigators on Vertex‐sponsored studies evaluating the use of ETI in patients with cystic fibrosis. G.C. has received speaker honoraria and research grant funding from Vertex Pharmaceuticals. D.S.U. has received speaker honoraria from Vertex Pharmaceuticals.

## GENERATIVE AI STATEMENT

No generative AI tools were used in the preparation of this manuscript.

## Data Availability

The data that support the findings of this study are available upon request. For the purpose of open access, the author(s) has (have) applied a Creative Commons Attribution (CC‐BY) license to any Author Accepted Manuscript version arising from this submission.

## 1

**TABLE A1:** Participant characteristics at enrolment to the e‐survey.

Characteristic	Value
Total survey participants (*n* (%))	
Person with CF	11 (37)
Parent or guardian of a person with CF	19 (63)
Age of all survey participants, mean (range) (years)	19 (6–61)
Geographical location (*n* (%))	
UK	9 (30.0)
Australia	18 (60.0)
France	1 (3.3)
Austria	1 (3.3)
Canada	1 (3.3)
CFTR genotype (*n* (%))	
Homozygous F508del	19 (63.3)
Heterozygous F508del	10 (33.3)
Other	1 (3.3)
CFTR modulator treatment (*n* (%))	
Kalydeco	1 (3.3)
Orkambi	1 (3.3)
Symkevi	1 (3.3)
Kaftrio/Trikafta	19 (63.3)
Not reported	4 (13.3)
None	4 (13.3)
School/employment (*n* (%))	
Student in school	19 (63.3)
Student in higher education	2 (6.7)
Student home‐schooled	1 (3.3)
Student but currently absent due to illness	1 (3.3)
Employed part‐time (less than 30 h per week)	3 (10)
Employed full‐time (30+h per week)	4 (13.3)
Physical activity behaviours, mean ± SD (min)	
Time spent walking over 7 days	307 ± 356
Time doing moderate PA over 7 days	274 ± 315
Time doing vigorous PA over 7 days	132 ± 144

**TABLE A2:** Responses to sweating frequency and characteristics during varying exercise intensities.

	All the time	Somewhat all the time	Neutral	Somewhat neutral	Never
Q1. Do you get sweaty during…					
Rest	6.7% (*n *= 2)	13.3% *(n *= 4)	10.0% (*n *= 3)	20.0% (*n *= 6)	50% *(n *= 15)
Low‐intensity exercise	10.0% (*n *= 3)	20.0% (*n *= 6)	36.7% (*n *= 11)	26.7% (*n *= 8)	6.7% (*n *= 2)
Moderate‐intensity exercise	23.3% (*n *= 7)	43.3% (*n *= 13)	16.7% (*n *= 5)	16.7% (*n *= 5)	0.0% (*n *= 0)
High‐intensity exercise	56.7% (*n *= 17)	30.0% (*n *= 9)	6.7% (*n *= 2)	3.3% (*n *= 1)	3.3% (*n *= 1)
Rest in heat	30.0% (*n *= 9)	23.3% (*n *= 7)	40.0% (*n *= 12)	3.3% (*n *= 1)	3.3% (*n *= 1)
Low‐intensity exercise in heat	36.7% (*n *= 11)	33.3% (*n *= 10)	20.0% (*n *= 6)	6.7% (*n *= 2)	3.3% (*n *= 1)
Moderate‐intensity exercise in heat	66.7% (*n *= 20)	26.7% (*n *= 8)	3.3% (*n *= 1)	3.3% (*n *= 1)	0.0% (*n *= 0)
High‐intensity exercise in heat	86.7% (*n *= 26)	10.0% (*n *= 3)	0.0% (*n *= 0)	0.0% (*n *= 0)	3.3% (*n *= 1)

Q2. Do you have salt on your skin during…					
Rest	3.3% (*n *= 1)	16.7% (*n *= 5)	13.3% (*n *= 4)	33.3% (*n *= 10)	33.3% (*n *= 10)
Low‐intensity exercise	16.7% (*n *= 5)	16.7% (*n *= 5)	10.0% (*n *= 3)	36.7% (*n *= 11)	20.0% (*n *= 6)
Moderate‐intensity exercise	26.7% (*n *= 8)	13.3% (*n *= 4)	20.0% (*n *= 6)	26.7% (*n *= 8)	13.3% (*n *= 4)
High‐intensity exercise	43.3% (*n *= 13)	16.7% (*n *= 5)	16.7% (*n *= 5)	10.0% (*n *= 5)	13.3% (*n *= 4)
Rest in heat	30.0% (*n *= 9)	20.0% (*n *= 6)	20.0% (*n *= 6)	16.7% (*n *= 5)	13.3% (*n *= 4)
Low‐intensity exercise in heat	33.3% (*n *= 10)	26.7% (*n *= 8)	16.7% (*n *= 5)	10.0% (*n *= 3)	13.3% (*n *= 4)
Moderate‐intensity exercise in heat	53.3% (*n *= 16)	20.0% (*n *= 6)	6.7% (*n *= 2)	6.7% (*n *= 2)	13.3% (*n *= 4)
High‐intensity exercise in heat	63.3% (*n *= 19)	10.0% (*n *= 3)	6.7% (*n *= 2)	10.0% (*n *= 3)	10.0% (*n *= 3)

**TABLE A3:** Perceived changes to sweating since initiating CFTR modulator therapies.

Question	Yes	No
On CFTR modulator treatment, have you noticed any changes with how sweaty you get since you started taking this?	40.0% (*n *= 12)	46.7% (*n *= 14)
On CFTR modulator treatment, have you noticed any changes with how salty your sweat gets since you started taking this?	43.3% (*n *= 13)	43.3% (*n *= 13)
**Example free‐text survey responses**		
‘Much less sweaty, less salt residue.’		
‘Feels like I get a bit less sweaty and there seems to be less salt in my sweat.’		
‘Although my child still sweats in certain situations, the amount he sweats has definitely decreased. So, on a regular night wrapped up in bed his pillows and hair would be soaked through but this has not happened since starting the triple therapy drug, even during exercise such as on his scooter for hours he does not sweat excessively.’		
‘No visible salt crystals or taste of salt on skin.’		
‘Was visibility sweaty in any warm weather before modulators.’		
‘Unaffected in hot environments with exercise, though less likely to feel sweaty at rest in a hot environment even when feeling hot. Overall, I feel I am sweating less.’		
‘My child does not have salty skin anymore.’		
‘It takes a lot for me to notice the salty taste on his skin from a kiss on his head now a days, that's definitely a big change I've noticed in regards to the triple therapy drug as he would taste very salty most days without doing much exercise beforehand.’		
‘He used to always be salty. He is less salty now at rest.’		

**TABLE A4:** Responses to self‐reported experiences of common symptoms of dehydration and/or heat‐related illness at rest and during exercise under varying climatic conditions.

Q1. Have you ever experienced…	At rest	During exercise	At rest in heat	During exercise in heat
Feeling tired	36.7% (*n *= 11)	63.3% (*n *= 19)	60.0% (*n *= 18)	83.3% (*n *= 25)
Cramps	23.3% (*n *= 7)	40.0% (*n *= 12)	10.0% (*n *= 3)	26.7% (*n *= 8)
Nausea	13.3% (*n *= 4)	16.7% (*n *= 5)	16.7% (*n *= 5)	30.0% (*n *= 9)
Dizziness	3.3% (*n *= 1)	30.0% (*n *= 9)	23.3% (*n *= 7)	30.0% (*n *= 9)
Thirst	56.7% (*n *= 17)	80.0% (*n *= 24)	86.7% (*n *= 26)	86.7% (*n *= 26)
Vomiting	6.7% (*n *= 2)	3.3% (*n *= 1)	6.7% (*n *= 2)	6.7% (*n *= 2)
Confusion	3.3% (*n *= 1)	10.0% (*n *= 3)	6.7% (*n *= 2)	16.7% (*n *= 5)
Muscle weakness	10.0% (*n *= 3)	30.0% (*n *= 9)	13.3% (*n *= 4)	36.7% (*n *= 11)
Heat sensations on head and neck	13.3% (*n *= 4)	26.7% (*n *= 8)	33.3% (*n *= 10)	40.0% (*n *= 12)
Chills	13.3% (*n *= 4)	0.0% (*n *= 0)	6.7% (*n *= 2)	0.0% (*n *= 0)
Feeling light headed	6.7% (*n *= 2)	16.7% (*n *= 5)	30.0% (*n *= 9)	40.0% (*n *= 12)
A dry mouth	26.7% (*n *= 8)	36.7% (*n *= 11)	36.7% (*n *= 11)	36.7% (*n *= 11)
Dark coloured, strong smelling urine	16.7% (*n *= 5)	10.0% (*n *= 3)	16.7% (*n *= 5)	26.7% (*n *= 8)
Passing urine less often than usual	0.0% (*n *= 0)	6.7% (*n *= 2)	6.7% (*n *= 2)	20.0% (*n *= 6)
Stomach cramps or pain	36.7% (*n *= 11)	23.3% (*n *= 7)	26.7% (*n *= 8)	26.7% (*n *= 8)
Constipation	30.0% (*n *= 9)	16.7% (*n *= 5)	20.0% (*n *= 6)	16.7% (*n *= 5)

**TABLE A5:** Perceived changes to heat‐related illness symptoms since initiating CFTR modulator therapies.

Question	Yes	No
On CFTR modulator treatment, have you noticed any changes with the dehydration or heat‐related illness symptoms you experience since you started taking this?	40.0% (*n *= 12)	46.7% (*n *= 14)
**Example free‐text survey responses**		
‘More energy, less tired. Less affected by 40° days. More thirst.’		
‘Less cramps, less tummy pain, less salt crystals on skin (TKA only)’		
‘I do not get headaches after exercise unless it has been extremely intense or all day e.g. hiking. I can take less salt and electrolytes for most activities. In the heat however I will still take as many as before Trikafta.’		
‘I feel that I get dehydrated quicker while on Trikafta. It feels like it dries all the moisture out of my body some days (along with the mucus).’		
‘No longer gets exhausted from hot weather alone.’		
‘Slightly less sweaty and heat reactive.’		
‘Not needing as many salt tablets.’		

**TABLE A6:** Participant‐reported hydration practices and daily water intake.

Q1. Do you follow guidelines for optimal hydration during…	Yes	No
Rest	53.3% (*n *= 16)	43.3% (*n *= 13)
Low‐intensity exercise	70.0% (*n *= 21)	30.0% (*n *= 9)
Moderate‐intensity exercise	70.0% (*n *= 21)	30.0% (*n *= 9)
High‐intensity exercise	70.0% (*n *= 21)	30.0% (*n *= 9)
Rest in heat	76.7% (*n *= 23)	23.3% (*n *= 7)
Low‐intensity exercise in heat	70.0% (*n *= 21)	26.7% (*n *= 8)
Moderate‐intensity exercise in heat	73.3% (*n *= 22)	26.7% (*n *= 8)
High‐intensity exercise in heat	73.3% (*n *= 22)	26.7% (*n *= 8)

Q2. On an average day my water intake would look like the following during…	No water	0–1 L	1–2 L	2–3 L	> 3 L of water
Rest	3.3% (*n *= 1)	73.3% (*n *= 22)	20.0% (*n *= 6)	3.3% (*n *= 1)	0.0% (*n *= 0)
Low‐intensity exercise	6.7% (*n *= 2)	56.7% (*n *= 17)	33.3% (*n *= 10)	3.3% (*n *= 1)	0.0% (*n *= 0)
Moderate‐intensity exercise	3.3% (*n *= 1)	40% (*n *= 12)	40.0% (*n *= 12)	13.3% (*n *= 4)	3.3% (*n *= 1)
High‐intensity exercise	6.7% (*n *= 2)	26.7% (*n *= 8)	33.3% (*n *= 10)	23.3% (*n *= 7)	10% (*n *= 3)
Rest in heat	0.0% (*n *= 0)	36.7% (*n *= 11)	46.7% (*n *= 14)	16.7% (*n *= 5)	0.0% (*n *= 0)
Low‐intensity exercise in heat	6.7% (*n *= 2)	23.3% (*n *= 7)	46.7% (*n *= 14)	16.7% (*n *= 5)	6.7% (*n *= 2)
Moderate‐intensity exercise in heat	6.7% (*n *= 2)	20.0% (*n *= 6)	36.7% (*n *= 11)	26.7% (*n *= 8)	10.0% (*n *= 3)
High‐intensity exercise in heat	6.7% (*n *= 2)	20.0% (*n *= 6)	30.0% (*n *= 9)	16.7% (*n *= 5)	26.7% (*n *= 8)

**TABLE A7:** Perceived changes to hydration status and fluid intake since initiating CFTR modulator therapies.

Q1. Since starting CFTR modulator treatment, have you noticed any changes with how much water you drink during…	Yes	No
Rest	10.0% (*n *= 3)	76.7% (*n *= 23)
Low‐intensity exercise	10.0% (*n *= 3)	76.7% (*n *= 23)
Moderate‐intensity exercise	13.3% (*n *= 4)	73.3% (*n *= 22)
High‐intensity exercise	10.0% (*n *= 3)	76.7% (*n *= 23)
Rest in heat	10.0% (*n *= 3)	76.7% (*n *= 23)
Low‐intensity exercise in heat	6.7% (*n *= 2)	80.0% (*n *= 24)
Moderate‐intensity exercise in heat	10.0% (*n *= 2)	76.7% (*n *= 23)
High‐intensity exercise in heat	10.0% (*n *= 3)	76.7% (*n *= 23)

Example free‐text survey responses		
‘Always thirsty. Hard to quench. Sliced cucumbers help with quench.’		
‘I feel as though I need to drink more water because I feel ‘drier’ in general on the Trikafta.’		
‘Slightly less water intake required/accepted by my child.’		
‘Less water.’		

**TABLE A8:** Participant‐reported knowledge, practices, and modulator‐related changes in salt intake, supplementation, and electrolyte use.

	Aware	Somewhat aware	Neutral	Somewhat unaware	Unaware
**Q1. I am aware that there are guidelines on salt intake and/or supplementation (tablets etc) for people with CF**:	53.3% (*n *= 16)	33.3% (*n *= 10)	3.3% (*n *= 1)	6.7% (*n *= 2)	3.3% (*n *= 1)
**Q2. I know how much salt my diet should include during…**	**Aware**	**Somewhat aware**	**Neutral**	**Somewhat unaware**	**Unaware**
Rest	40% (*n *= 12)	16.7% (*n *= 5)	16.7% (*n *= 5)	13.3% (*n *= 4)	13.3% (*n *= 4)
Low‐intensity exercise	40% (*n *= 12)	16.7% (*n *= 5)	13.3% (*n *= 4)	13.3% (*n *= 4)	16.7% (*n *= 5)
Moderate‐intensity exercise	36.7% (*n *= 11)	20% (*n *= 6)	13.3% (*n *= 4)	13.3% (*n *= 4)	16.7% (*n *= 5)
High‐intensity exercise	36.7% (*n *= 11)	23.3% (*n *= 7)	13.3% (*n *= 4)	10% (*n *= 3)	16.7% (*n *= 5)
Rest in heat	46.7% (*n *= 14)	10% (*n *= 3)	16.7% (*n *= 5)	13.3% (*n *= 4)	13.3% (*n *= 4)
Low‐intensity exercise in heat	40% (*n *= 12)	20% (*n *= 6)	13.3% (*n *= 4)	13.3% (*n *= 4)	13.3% (*n *= 4)
Moderate‐intensity exercise in heat	40% (*n *= 12)	20% (*n *= 6)	13.3% (*n *= 4)	13.3% (*n *= 4)	13.3% (*n *= 4)
High‐intensity exercise in heat	40% (*n *= 12)	20% (*n *= 6)	16.7% (*n *= 5)	10% (*n *= 3)	13.3% (*n *= 4)
**Q3. Do you crave salt or salty foods during or after…**	**All the time**	**Somewhat all the time**	**Neutral**	**Somewhat neutral**	**Never**
Rest	10% (*n *= 3)	16.7% (*n *= 5)	36.7% (*n *= 11)	6.7% (*n *= 2)	26.7% (*n *= 8)
Low‐intensity exercise	10% (*n *= 3)	16.7% (*n *= 5)	43.3% (*n *= 13)	3.3% (*n *= 1)	26.7% (*n *= 8)
Moderate‐intensity exercise	10% (*n *= 3)	26.7% (*n *= 8)	36.7% (*n *= 11)	0% (*n *= 0)	26.7% (*n *= 8)
High‐intensity exercise	20% (*n *= 6)	23.3% (*n *= 7)	23.3% (*n *= 7)	3.3% (*n *= 1)	26.7% (*n *= 8)
Rest in heat	13.3% (*n *= 4)	23.3% (*n *= 7)	40% (*n *= 12)	3.3% (*n *= 1)	20% (*n *= 6)
Low‐intensity exercise in heat	13.3% (*n *= 4)	26.7% (*n *= 8)	33.3% (*n *= 10)	3.3% (*n *= 1)	23.3% (*n *= 7)
Moderate‐intensity exercise in heat	10% (*n *= 3)	36.7% (*n *= 11)	26.7% (*n *= 8)	0% (*n *= 0)	26.7% (*n *= 8)
High‐intensity exercise in heat	20% (*n *= 6)	26.7% (*n *= 8)	23.3% (*n *= 7)	3.3% (*n *= 1)	26.7% (*n *= 8)
**Q4. Since starting CFTR modulator treatment, have you noticed any changes with how much salt you crave**			**Yes**		**No**
			23.3% (*n *= 7)		63.3% (*n *= 19)
**Q5. If yes, did you crave salt or salty foods before starting a modulator drug during or after…**	**All the time**	**Somewhat all the time**	**Neutral**	**Somewhat neutral**	**Never**
Rest	3.3% (*n *= 1)	20% (*n *= 6)	13.3% (*n *= 4)	0% (*n *= 0)	13.3% (*n *= 4)
Low‐intensity exercise	3.3% (*n *= 1)	13.3% (*n *= 4)	16.7% (*n *= 5)	0% (*n *= 0)	16.7% (*n *= 5)
Moderate‐intensity exercise	10% (*n *= 3)	6.7% (*n *= 2)	16.7% (*n *= 5)	0% (*n *= 0)	16.7% (*n *= 5)
High‐intensity exercise	13.3% (*n *= 4)	6.7% (*n *= 2)	16.7% (*n *= 5)	3.3% (*n *= 1)	10% (*n *= 3)
Rest in heat	6.7% (*n *= 2)	13.3% (*n *= 4)	20% (*n *= 6)	3.3% (*n *= 1)	6.7% (*n *= 2)
Low‐intensity exercise in heat	6.7% (*n *= 2)	13.3% (*n *= 4)	16.7% (*n *= 5)	3.3% (*n *= 1)	10% (*n *= 3)
Moderate‐intensity exercise in heat	10% (*n *= 3)	10% (*n *= 3)	16.7% (*n *= 5)	3.3% (*n *= 1)	10% (*n *= 3)
High‐intensity exercise in heat	16.7% (*n *= 5)	3.3% (*n *= 1)	16.7% (*n *= 5)	3.3% (*n *= 1)	10% (*n *= 3)
**Q6. Do you take salt supplements during…**			**Yes**		
Rest			20% (*n *= 6)		
Exercise			20% (*n *= 6)		
Rest in heat			26.7% (*n *= 8)		
Exercise in heat			30% (*n *= 9)		
**Q7. On CFTR modulator treatment, have you made any changes to the salt supplements you take during…**			**Yes**		**No**
Rest			10% (*n *= 3)		53.3% (*n *= 16)
Exercise			10% (*n *= 3)		50% (*n *= 15)
Rest in heat			10% (*n *= 3)		50% (*n *= 15)
Exercise in heat			10% (*n *= 3)		50% (*n *= 15)
**Q8. Do you add salt to your food during…**			**Yes**		
Rest			23.3% (*n *= 7)		
Exercise			26.7% (*n *= 8)		
Rest in heat			33.3% (*n *= 10)		
Exercise in heat			33.3% (*n *= 10)		
**Q9. Average salt added to food during…**			**Mean **± **SD (g)**		**Range (g)**
Rest			0.4 ± 1.3		(0–6.3)
Low‐intensity exercise			0.5 ± 1.6		(0–6.3)
Moderate‐intensity exercise			0.7 ± 1.7		(0–6.3)
High‐intensity exercise			0.8 ± 1.8		(0–6.3)
Rest in heat			0.7 ± 1.7		(0–6.3)
Low‐intensity exercise in heat			0.9 ± 2.0		(0–6.6)
Moderate‐intensity exercise in heat			1.0 ± 2.4		(0–9.3)
High‐intensity exercise in heat			1.1 ± 2.5		(0–9.6)
**Q10. On CFTR modulator treatment, have you made any changes to the salt you add to your food?**			**Yes**		**No**
			23.3% (*n *= 7)		63.3% (*n *= 19)
**Q11. Do you consume electrolyte drinks during…**			**Yes**		
Rest			3.3% (*n *= 1)		
Exercise			40% (*n *= 12)		
Rest in heat			20% (*n *= 6)		
Exercise in heat			33.3% (*n *= 10)		
**Q12. Average salt consumed via electrolytes drinks…**			**Mean **± **SD (g)**		**Range (g)**
Rest			0.0 ± 0.1		(0–0.6)
Low‐intensity exercise			0.0 ± 0.1		(0–0.6)
Moderate‐intensity exercise			0.1 ± 0.3		(0–1.2)
High‐intensity exercise			0.4 ± 0.7		(0–3.6)
Rest in heat			0.3 ± 0.7		(0–3.7)
Low‐intensity exercise in heat			0.2 ± 0.7		(0–3.7)
Moderate‐intensity exercise in heat			0.3 ± 0.7		(0–3.7)
High‐intensity exercise in heat			0.4 ± 0.7		(0–3.7)

## References

[eph70445-bib-0001] AghaKouchak, A. , Chiang, F. , Huning, L. S. , Love, C. A. , Mallakpour, I. , Mazdiyasni, O. , Moftakhari, H. , Papalexiou, S. M. , Ragno, E. , & Sadegh, M. (2020). Climate extremes and compound hazards in a warming world. Annual Review of Earth and Planetary Sciences, 48(1), 519–548.

[eph70445-bib-0002] Baker, L. B. (2019). Physiology of sweat gland function: The roles of sweating and sweat composition in human health. Temperature, 6(3), 211–259. . 10.1080/23328940.2019.1632145 PMC6773238 31608304

[eph70445-bib-0003] Ballestero, Y. , Hernandez, M. I. , Rojo, P. , Manzanares, J. , Nebreda, V. , Carbajosa, H. , Infante, E. , & Baro, M. (2006). Hyponatremic dehydration as a presentation of cystic fibrosis. Pediatric Emergency Care, 22(11), 725–727. 17110865 10.1097/01.pec.0000245170.31343.bb

[eph70445-bib-0004] Bar‐Or, O. , Hay, J. A. , Ward, D. , Blimkie, C. , MacDougall, J. , & Wilson, W. (1992). Voluntary dehydration and heat intolerance in cystic fibrosis. Lancet, 339(8795), 696–699. 1347582 10.1016/0140-6736(92)90597-v

[eph70445-bib-0005] Berg, R. A. , Jensen‐Fangel, S. , Bandulik, S. , Warth, R. , Sørensen, M. V. , Jeppesen, M. , & Leipziger, J. (2025). Elexacaftor/Tezacaftor/Ivacaftor corrects salt‐wasting in cystic fibrosis. Journal of Cystic Fibrosis, 25(1), 32–37. 41365775 10.1016/j.jcf.2025.12.006

[eph70445-bib-0006] Blatteis, C. M. (2012). Age‐dependent changes in temperature regulation–a mini review. Gerontology, 58(4), 289–295. 22085834 10.1159/000333148

[eph70445-bib-0007] Borg, G. A. (1982). Psychophysical bases of perceived exertion. Medicine and Science in Sports and Exercise, 14(5), 377–381. 7154893

[eph70445-bib-0008] Brown, M. B. , McCarty, N. A. , & Millard‐Stafford, M. (2011). High‐sweat Na+ in cystic fibrosis and healthy individuals does not diminish thirst during exercise in the heat. American Journal of Physiology‐Regulatory, Integrative and Comparative Physiology, 301(4), R1177–R1185. 21813870 10.1152/ajpregu.00551.2010 PMC3197340

[eph70445-bib-0009] Clayton, L. J. , Shepherd, A. I. , Corbett, J. O. , Perissiou, M. , Connett, G. , Legg, J. , Allenby, M. , Daniels, T. , Urquhart, D. S. , Mackintosh, K. A. , McNarry, M. A. , & Saynor, Z. L. (2025). Cardiovascular function in people with cystic fibrosis on Elexacaftor/Tezacaftor/Ivacaftor: A cross‐sectional, observational, single‐centre study. Journal of Cystic Fibrosis, 24(4), 801–808. 39956715 10.1016/j.jcf.2025.02.001

[eph70445-bib-0010] Coris, E. E. , Walz, S. M. , Duncanson, R. , Ramirez, A. M. , & Roetzheim, R. G. (2006). Heat illness symptom index (HISI): A novel instrument for the assessment of heat illness in athletes. Southern Medical Journal, 99(4), 340–345. 16634241 10.1097/01.smj.0000209285.96906.0f

[eph70445-bib-0011] Craig, C. L. , Marshall, A. L. , Sjöström, M. , Bauman, A. E. , Booth, M. L. , Ainsworth, B. E. , Pratt, M. , Ekelund, U. , Yngve, A. , Sallis, J. F. , & Oja, P. (2003). International physical activity questionnaire: 12‐country reliability and validity. Medicine & Science in Sports & Exercise, 35(8), 1381–1395. 12900694 10.1249/01.MSS.0000078924.61453.FB

[eph70445-bib-0012] Cramer, M. N. , & Jay, O. (2014). Selecting the correct exercise intensity for unbiased comparisons of thermoregulatory responses between groups of different mass and surface area. Journal of Applied Physiology, 116(9), 1123–1132. 24505102 10.1152/japplphysiol.01312.2013

[eph70445-bib-0013] Cramer, M. N. , & Jay, O. (2015). Explained variance in the thermoregulatory responses to exercise: The independent roles of biophysical and fitness/fatness‐related factors. Journal of Applied Physiology, 119(9), 982–989. 26316511 10.1152/japplphysiol.00281.2015 PMC4628991

[eph70445-bib-0014] di Sant'Agnese, P. A. , Darling, R. C. , Perera, G. A. , & Shea, E. (1953). Abnormal electrolyte composition of sweat in cystic fibrosis of the pancreas: Clinical significance and relationship to the disease. Pediatrics, 12(5), 549–563. 13111855

[eph70445-bib-0015] Du Bois, D. (1916). A formula to estimate the approx‐imate surface area if height and weight be known. Archives of Internal Medicine, 17, 863–871.

[eph70445-bib-0016] Emrich, H. , Stoll, E. , Friolet, B. , Colombo, J. , Richterich, R. , & Rossi, E. (1968). Sweat composition in relation to rate of sweating in patients with cystic fibrosis of the pancreas. Pediatric Research, 2(6), 464–478. 5727917 10.1203/00006450-196811000-00004

[eph70445-bib-0017] Frost, F. , Nazareth, D. , Fauchier, L. , Wat, D. , Shelley, J. , Austin, P. , Walshaw, M. J. , & Lip, G. Y. H. (2023). Prevalence, risk factors and outcomes of cardiac disease in cystic fibrosis: A multinational retrospective cohort study. European Respiratory Journal, 62(4), 2300174. 37474158 10.1183/13993003.00174-2023 PMC10600351

[eph70445-bib-0018] Gagnon, D. , Jay, O. , Lemire, B. , & Kenny, G. P. (2008). Sex‐related differences in evaporative heat loss: The importance of metabolic heat production. European Journal of Applied Physiology, 104(5), 821–829. 18677506 10.1007/s00421-008-0837-0

[eph70445-bib-0019] Gruet, M. , Saynor, Z. L. , Urquhart, D. S. , & Radtke, T. (2022). Rethinking physical exercise training in the modern era of cystic fibrosis: A step towards optimising short‐term efficacy and long‐term engagement. Journal of Cystic Fibrosis, 21(2), e83–e98. 34493444 10.1016/j.jcf.2021.08.004

[eph70445-bib-0020] Heijerman, H. G. M. , McKone, E. F. , Downey, D. G. , Van Braeckel, E. , Rowe, S. M. , Tullis, E. , Mall, M. A. , Welter, J. J. , Ramsey, B. W. , McKee, C. M. , Marigowda, G. , Moskowitz, S. M. , Waltz, D. , Sosnay, P. R. , Simard, C. , Ahluwalia, N. , Xuan, F. , Zhang, Y. , ,… Taylor‐Cousar, J. L. , VX17‐445‐103 Trial Group . (2019). Efficacy and safety of the elexacaftor plus tezacaftor plus ivacaftor combination regimen in people with cystic fibrosis homozygous for the F508del mutation: A double‐blind, randomised, phase 3 trial. Lancet, 394(10212), 1940–1948. 31679946 10.1016/S0140-6736(19)32597-8 PMC7571408

[eph70445-bib-0021] Hulzebos, H. , Werkman, M. , van Brussel, M. , & Takken, T. (2012). Towards an individualized protocol for workload increments in cardiopulmonary exercise testing in children and adolescents with cystic fibrosis. Journal of Cystic Fibrosis, 11(6), 550–554. 22704761 10.1016/j.jcf.2012.05.004

[eph70445-bib-0022] Jay, O. , & Cramer, M. (2016). Biophysical aspects of human thermoregulation during heat stress. Autonomic Neuroscience: Basic & Clinical, 196, 3–13. 26971392 10.1016/j.autneu.2016.03.001

[eph70445-bib-0023] Kessler, W. R. , & Andersen, D. H. (1951). Heat prostration in fibrocystic disease of the pancreas and other conditions. Pediatrics, 8(5), 648–656. 14891321

[eph70445-bib-0024] Kriemler, S. , Wilk, B. , Schurer, W. , Wilson, W. M. , & Bar‐Or, O. (1999). Preventing dehydration in children with cystic fibrosis who exercise in the heat. Medicine and Science in Sports and Exercise, 31(6), 774–779. 10378902 10.1097/00005768-199906000-00003

[eph70445-bib-0025] Lopes‐Pacheco, M. (2020). CFTR modulators: The changing face of cystic fibrosis in the era of precision medicine. Frontiers in Pharmacology, 10, 1662. 32153386 10.3389/fphar.2019.01662 PMC7046560

[eph70445-bib-0026] Malta, D. , Petersen, K. S. , Johnson, C. , Trieu, K. , Rae, S. , Jefferson, K. , Santos, J. A. , Wong, M. M. Y. , Raj, T. S. , Webster, J. , Campbell, N. R. C. , & Arcand, J. (2018). High sodium intake increases blood pressure and risk of kidney disease. From the Science of Salt: A regularly updated systematic review of salt and health outcomes. Journal of Clinical Hypertension, 20(12), 1654–1665. 30402970 10.1111/jch.13408 PMC8030856

[eph70445-bib-0027] McKone, E. F. , Borowitz, D. , Drevinek, P. , Griese, M. , Konstan, M. W. , Wainwright, C. , Ratjen, F. , Sermet‐Gaudelus, I. , Plant, B. , Munck, A. , Jiang, Y. , Gilmartin, G. , & Davies, J. C. (2014). Long‐term safety and efficacy of ivacaftor in patients with cystic fibrosis who have the Gly551Asp‐CFTR mutation: A phase 3, open‐label extension study (PERSIST). Lancet Respiratory medicine, 2(11), 902–910. 25311995 10.1016/S2213-2600(14)70218-8

[eph70445-bib-0028] Middleton, P. G. , Mall, M. A. , Dřevínek, P. , Lands, L. C. , McKone, E. F. , Polineni, D. , Ramsey, B. W. , Taylor‐Cousar, J. L. , Tullis, E. , & Vermeulen, F. (2019). Elexacaftor–tezacaftor–ivacaftor for cystic fibrosis with a single Phe508del allele. New England Journal of Medicine, 381(19), 1809–1819. 31697873 10.1056/NEJMoa1908639 PMC7282384

[eph70445-bib-0029] NOAA National Centres for Environmental Information . (2023). Monthly Global Climate Report for Annual 2022. Online: https://www.ncei.noaa.gov/access/monitoring/monthly‐report/global/202213<./bib>

[eph70445-bib-0030] Orenstein, D. M. , Henke, K. G. , Costill, D. L. , Doershuk, C. F. , Lemon, P. J. , & Stern, R. C. (1983). Exercise and heat stress in cystic fibrosis patients. Pediatric Research, 17(4), 267–269. 6343989 10.1203/00006450-198304000-00007

[eph70445-bib-0031] Orenstein, D. M. , Henke, K. G. , & Green, C. G. (1984). Heat acclimation in cystic fibrosis. Journal of Applied Physiology, 57(2), 408–412. 6381439 10.1152/jappl.1984.57.2.408

[eph70445-bib-0032] Périard, J. D. , Eijsvogels, T. M. , & Daanen, H. A. (2021). Exercise under heat stress: Thermoregulation, hydration, performance implications, and mitigation strategies. Physiological Reviews, 101(4), 1873–1979. 33829868 10.1152/physrev.00038.2020

[eph70445-bib-0033] Poore, S. , Berry, B. , Eidson, D. , McKie, K. T. , & Harris, R. A. (2013). Evidence of vascular endothelial dysfunction in young patients with cystic fibrosis. Chest, 143(4), 939–945. 23099448 10.1378/chest.12-1934

[eph70445-bib-0034] Quinton, P. M. (1983). Chloride impermeability in cystic fibrosis. Nature, 301(5899), 421–422. 6823316 10.1038/301421a0

[eph70445-bib-0035] Radtke, T. , Smith, S. , Nevitt, S. J. , Hebestreit, H. , & Kriemler, S. (2022). Physical activity and exercise training in cystic fibrosis. Cochrane Database of Systematic Reviews, 8(8), CD002768. 35943025 10.1002/14651858.CD002768.pub5 PMC9361297

[eph70445-bib-0036] Rajan, V. , Varghese, B. , van Leeuwen, T. G. , & Steenbergen, W. (2009). Review of methodological developments in laser Doppler flowmetry. Lasers in Medical Science, 24(2), 269–283. 18236103 10.1007/s10103-007-0524-0

[eph70445-bib-0037] Ramanathan, N. (1964). A new weighting system for mean surface temperature of the human body. Journal of Applied Physiology, 19(3), 531–533. 14173555 10.1152/jappl.1964.19.3.531

[eph70445-bib-0038] Ramsey, B. W. , Davies, J. , McElvaney, N. G. , Tullis, E. , Bell, S. C. , Dřevínek, P. , Griese, M. , McKone, E. F. , Wainwright, C. E. , & Konstan, M. W. (2011). A CFTR potentiator in patients with cystic fibrosis and the G551D mutation. New England Journal of Medicine, 365(18), 1663–1672. 22047557 10.1056/NEJMoa1105185 PMC3230303

[eph70445-bib-0039] Rodriguez‐Miguelez, P. , Thomas, J. , Seigler, N. , Crandall, R. , McKie, K. T. , Forseen, C. , & Harris, R. A. (2016). Evidence of microvascular dysfunction in patients with cystic fibrosis. American Journal of Physiology. Heart and Circulatory Physiology, 310(11), H1479–H1485. 27084387 10.1152/ajpheart.00136.2016 PMC4935515

[eph70445-bib-0040] Saint‐Criq, V. , & Gray, M. A. (2017). Role of CFTR in epithelial physiology. Cellular and Molecular Life Sciences, 74(1), 93–115. 27714410 10.1007/s00018-016-2391-y PMC5209439

[eph70445-bib-0041] Sawka, M. N. , Burke, L. M. , Eichner, E. R. , Maughan, R. J. , Montain, S. J. , & Stachenfeld, N. S. (2009). Exercise and fluid replacement. Medicine and Science in Sports and Exercise, 39(2), 377–390. 10.1249/mss.0b013e31802ca597 17277604

[eph70445-bib-0042] Sawka, M. N. , Wenger, C. B. , & Pandolf, K. B. (2010). Thermoregulatory responses to acute exercise‐heat stress and heat acclimation. Comprehensive Physiology, 1994(11S14), 157–185.

[eph70445-bib-0043] Sawka, M. N. , Young, A. J. , Latzka, W. A. , Neufer, P. D. , Quigley, M. D. , & Pandolf, K. B. (1992). Human tolerance to heat strain during exercise: Influence of hydration. Journal of Applied Physiology, 73(1), 368–375. 1506393 10.1152/jappl.1992.73.1.368

[eph70445-bib-0044] American Thoracic Society . (2019). Standardization of spirometry 2019 update an official American Thoracic Society and European Respiratory Society technical statement. American Journal of Respiratory and Critical Care Medicine, 200(8), E70–E88. 31613151 10.1164/rccm.201908-1590ST PMC6794117

[eph70445-bib-0045] Southern, K. W. , Addy, C. , Bell, S. C. , Bevan, A. , Borawska, U. , Brown, C. , Burgel, P.‐R. , Button, B. , Castellani, C. , Chansard, A. , Chilvers, M. A. , Davies, G. , Davies, J. C. , De Boeck, K. , Declercq, D. , Doumit, M. , Drevinek, P. , Fajac, I. , Gartner, S. , … van Koningsbruggen‐Rietschel, S. (2024). Standards for the care of people with cystic fibrosis; establishing and maintaining health. Journal of Cystic Fibrosis, 23(1), 12–28. 38129255 10.1016/j.jcf.2023.12.002

[eph70445-bib-0046] Taylor, N. A. , & Machado‐Moreira, C. A. (2013). Regional variations in transepidermal water loss, eccrine sweat gland density, sweat secretion rates and electrolyte composition in resting and exercising humans. Extreme Physiology & Medicine, 2(1), 4. 23849497 10.1186/2046-7648-2-4 PMC3710196

[eph70445-bib-0047] Taylor‐Cousar, J. L. , Munck, A. , McKone, E. F. , van der Ent, C. K. , Moeller, A. , Simard, C. , Wang, L. T. , Ingenito, E. P. , McKee, C. , Lu, Y. , Lekstrom‐Himes, J. , & Elborn, J. S. (2017). Tezacaftor–ivacaftor in patients with cystic fibrosis homozygous for Phe508del. New England Journal of Medicine, 377(21), 2013–2023. 29099344 10.1056/NEJMoa1709846

[eph70445-bib-0048] Tipton, M. , Pinho‐Gomes, A.‐C. , Bailey, D. , Bills, V. , Dearman, A. , Foster, J. , Havenith, G. , Howarth, G. , Montgomery, H. , & Mulcahy, E. (2023). Red Alert: Developing a Humancentred National Heat Resilience Strategy. Age, 16, 19.

[eph70445-bib-0049] Williams, A. , McKiernan, J. , & Harris, F. (1976). Heat prostration in children with cystic fibrosis. British Medical Journal, 2(6030), 297.1–29297. 10.1136/bmj.2.6030.297 PMC1687938 953570

[eph70445-bib-0050] Wilschanski, M. , Munck, A. , Carrion, E. , Cipolli, M. , Collins, S. , Colombo, C. , Declercq, D. , Hatziagorou, E. , Hulst, J. , Kalnins, D. , Katsagoni, C. N. , Mainz, J. G. , Ribes‐Koninckx, C. , Smith, C. , Smith, T. , Van Biervliet, S. , & Chourdakis, M. (2024). ESPEN‐ESPGHAN‐ECFS guideline on nutrition care for cystic fibrosis. Clinical Nutrition, 43(2), 413–445. 38169175 10.1016/j.clnu.2023.12.017

[eph70445-bib-0051] Zemanick, E. T. , Taylor‐Cousar, J. L. , Davies, J. , Gibson, R. L. , Mall, M. A. , McKone, E. F. , McNally, P. , Ramsey, B. W. , Rayment, J. H. , Rowe, S. M. , Tullis, E. , Ahluwalia, N. , Chu, C. , Ho, T. , Moskowitz, S. M. , Noel, S. , Tian, S. , Waltz, D. , Weinstock, T. G. , … McColley, S. A. (2021). A phase 3 open‐label study of Elexacaftor/Tezacaftor/Ivacaftor in children 6 through 11 years of age with cystic fibrosis and at least one F508del allele. American Journal of Respiratory and Critical Care Medicine, 203(12), 1522–1532. 33734030 10.1164/rccm.202102-0509OC PMC8483230

[eph70445-bib-0052] Zhang, Y. , & Zhao, R. (2008). Overall thermal sensation, acceptability and comfort. Building & Environment, 43(1), 44–50.

